# E3 ligase TRIM22 promotes melanoma proliferation by regulating cell cycle progression through K63-linked ubiquitination of p21

**DOI:** 10.1038/s41598-025-06348-4

**Published:** 2025-07-01

**Authors:** Chen-xing Jin, Ting-ze Feng, Xiang Ji, Yan-song Liu, He-nan Qin, Yi-bin Teng, Chibuzo Sampson, Tian Xia, Hai-long Piao, Ji-wei Liu

**Affiliations:** 1 Department of Oncology, The First Affiliated Hospital of Dalian Medical University, DalianLiaoning, 116011 China; 2 Dalian Institute of Chemical Physics, Chinese Academy of Sciences, DalianLiaoning, 116023 China; 3 Department of Anesthesiology, Obstetrics and Gynecology Hospital of Fudan University, Shanghai, 200011 China; 4 University of Chinese Academy of Sciences, Beijing, 100049 China

**Keywords:** Ubiquitination, TRIM22, p21, Cell cycle, Proliferation, Cancer, Cell biology, Molecular biology

## Abstract

Melanoma, a highly aggressive skin cancer with limited therapeutic options, demonstrates poor prognosis in advanced stages. Tripartite motif-containing 22 (TRIM22), an E3 ubiquitin ligase of the tripartite motif (TRIM) family, is implicated in tumorigenesis, but its working mechanism remains poorly understood in melanoma. In this study, we found that expression of TRIM22 was abnormally upregulated in melanoma tissues, correlating with tumor stages. Functional analysis demonstrated that TRIM22 promoted melanoma cell proliferation in vitro. Furthermore, we found that in malignant melanoma, TRIM22 expression is negatively correlated to the level of p21, an inhibitor of cell cycle. With quantitative real-time PCR (qRT-PCR) assay and cycloheximide (CHX) treatment, we confirmed that TRIM22 suppressed p21 expression at protein level. Via S-Protein pull-down assay, we found that p21 could interact with TRIM22 at the SPRY domain. A ubiquitination assay proved that TRIM22 promoted the K63-linked ubiquitination of p21, and thereby induced p21 degradation through the proteasome pathway to accelerate cell cycle progression. Moreover, we discovered that overexpression of TRIM22 could not bring further boost of cell proliferation in p21 knockdown melanoma cells, indicating an epistatic role of p21 to TRIM22. Overall, our findings elucidated that TRIM22 acted as an E3 ligase targeting p21 for degradation to promote melanoma progression, which improved the understanding of TRIM22 function and provided more clues for developing TRIM22 as a potential target for malignant melanoma treatment.

## Introduction

Melanoma is a malignancy arising from the transformation of the melanocytes^1^. Cutaneous melanoma is the most common type of malignant melanoma, accounting for about 90%, with the melanoma of mucosal and uveal origin occurring rarely^2^. The incidence of melanoma is still increasing by 2%-3% every year, a rate faster than almost any other malignancy^3,4^. Benefiting from the advent of immune-checkpoint inhibitors (ICIs) and targeted therapies, the situation of melanoma treatment has undergone a dramatic improvement in the past decade^5–7^. Although current treatment options provide a clinical benefit, the late stages (Stage III and IV) of melanoma are associated with a precipitous drop in median 5-year survival to about 30%^3,8^. Moreover, despite the promising results of ICIs in melanoma, approximately half of patients do not derive long-lasting benefit^9^. In addition, combined agents also develop acquired resistance at a median of 9–11 months in patients receiving targeted therapy^10^. The challenge of managing patients who develop resistance to existing therapies remains a significant clinical concern^11,12^. Therefore, it is imperative to explore the mechanisms underpinning melanoma progression and to seek comprehensive and effective approaches for treating melanoma.

Ubiquitination, a pivotal posttranslational modification (PTM) of protein, plays fundamental roles in regulating protein stability and activity^13^. Ubiquitination is a multistep process mediated by the sequential activities of ubiquitin-activating enzyme (E1), ubiquitin-conjugating enzyme (E2), and ubiquitin ligase (E3)^14^. Ubiquitination is crucial in regulating tumor behaviors, such as proliferation, apoptosis, invasion, metastasis, angiogenesis, reprogrammed metabolism, and immune escape^15^. Ubiquitin contains seven lysine residues (K6, K11, K27, K29, K33, K48, and K63) and one N-terminal methionine (M1). These residues can serve as the docking sites for the formation of additional ubiquitin chains. Notably, the polyubiquitination of target proteins results in distinct functional consequences, which are contingent upon the specific lysine residue of ubiquitin involved in the linkage^16^. K48-linked polyubiquitylation is well-known as a symbol of targeting proteins for degradation by 26S proteasome^17^. K63-linked polyubiquitination has been associated with numerous cellular events that do not necessarily rely on degradative signaling via the proteasome^18^. E3 ligases are considered the most crucial enzymes in the ubiquitination process due to their role in conferring substrate specificity. Several studies have shown the critical roles of E3 ligases in melanoma^19–21^. For example, downregulated ring finger protein 128 (RNF128) activates Wnt signaling to promote epithelial-mesenchymal transition and stemness by ubiquitinating CD44 and cortactin for degradation ^22^. F-box only protein 32 (FBXO32) is known to promote melanoma cell migration and proliferation^23^. In addition, tumor necrosis factor receptor-associated factor 6 (TRAF6) promotes tumor immune escape by enhancing the expression of programmed death-ligand 1 (PD-L1) through stabilizing Yes-associated protein 1 (YAP1)^24^. The E3 ligase ITCH, also named atrophin-1 interacting protein 4 ^25^, can directly interact with BRAF and promote its K27-linked polyubiquitination, resulting in the sustained activation of BRAF as well as its downstream signaling cascade^26^. In MAPK inhibitor- adapted melanoma cells, ITCH can downregulate the immunosuppressor, PD-L1 levels via ubiquitin-directed lysosomal degradation, which can enhance tumor rejection by activating cytolytic CD8^+^ T cells^27^. These examples demonstrate the diverse and significant functions of E3 ligases in melanoma, highlighting their multifaceted roles in various cellular processes related to tumor development. Thus, E3 ligases, as critical players in melanoma progression and therapy resistance, represent ideal therapeutic candidates for melanoma.

The tripartite motif (TRIM) family proteins were first known for their involvement in innate immunity as pathogen-recognition effectors and regulators in transcriptional pathways^28^. Most of the TRIM family proteins, which function as E3 ligases, are involved in various physiological and pathological processes, including signal transduction, cell proliferation, and apoptosis^29,30^. All TRIM family proteins contain an N-terminal RING finger domain, followed by one or two B-box motifs, an associated coiled-coil domain and a highly variable carboxyl-terminal domain^31^. Over the last decade, many studies have uncovered the roles of TRIM family members in cancer initiation, progression, and therapy resistance, in which TRIM family proteins exhibit both oncogenic and tumor-suppression functions in different human cancer types^31–35^. TRIM22 was first identified as a new interferon (IFN)-induced TRIM family protein and has been implicated in regulating the tumorigenesis and progression in various cancers^36,37^. In glioblastoma (GBM), TRIM22 has been identified as an activator of NF-$\kappa$ B signaling by respectively promoting K48- and K63-linked ubiquitination of I kappa B protein alpha (IκBα) and I kappa B kinase gamma (IKK $\gamma$), which drive tumor growth and progression^38^. TRIM22 has also been shown to regulate MAPK signaling in GBM by mediating K48-linked ubiquitination and degradation of Raf-1 and the transcriptional regulation of sphingosine kinase 2 (SPHK2), thereby promoting GBM proliferation^39^. High expression of TRIM22 in esophageal squamous cell carcinomas (ESCC), correlates positively with cell proliferation, metastasis and invasion^40^. In contrast to its tumor-promoting role in GBM and ESCC, a distinct function of TRIM22 as a tumor suppressor has been identified in some cancers. It is found that TRIM22 inhibits osteosarcoma (OS) progression by promoting proteasomal degradation of nuclear factor erythroid 2-related factor 2 (NRF2) independent of its canonical regulator Kelch-like ECH-associated protein 1 (KEAP1), thereby activating ROS/AMPK/mTOR/autophagy signaling for autophagic cell death^41^. TRIM22 also inhibits the proliferation and invasion of breast cancer (BC) through ubiquitination and degradation of copper chaperone for superoxide dismutase (CCS)^42^. Moreover, TRIM22 has recently been identified as a negative regulator of major histocompatibility complex class II (MHC-II) expression, indicating a direct immunosuppressive role of considerable interest in checkpoint blockade cancer immunotherapy^43^. While TRIM22 is implicated in diverse cellular functions and plays distinct role in different cancers, its contribution to melanoma progression and the underlying mechanisms remains largely underexplored.

In this study, we aimed to elucidate the role of TRIM22 in melanoma and the underlying mechanism that drives melanoma progression. We demonstrated that TRIM22 expression was significantly upregulated in melanoma, which positively correlated with melanoma progression and patient tumor stage. Moreover, TRIM22 promoted cell cycle progression and proliferation of melanoma cells in vitro. Mechanistically, we identified a key cell cycle inhibitor, p21, as an unreported target of TRIM22. Through physical interaction, TRIM22 promoted p21 K63-linked ubiquitination and subsequent proteasomal degradation in melanoma. These findings unravel a previously unrecognized function of TRIM22 in malignant melanoma, where it regulates cell cycle progression by targeting p21 for ubiquitination and degradation. This novel insight contributes to a more comprehensive understanding of the working mechanism and oncogenic functions of TRIM22, and a new regulation mechanism of p21 and cell cycle in melanoma. Taken together, our study identifies TRIM22 as an oncogenic driver that could be considered a potential therapeutic target for melanoma treatment.

## Materials and methods

### Cell lines and culture conditions

Human melanoma cell line A375 (Stem Cell Bank, Chinese Academy of Sciences) and embryonic kidney 293T cells (HEK293T) (American Type Culture Collection, United States) were cultured in Dulbecco’s Modified Eagle’s medium (DMEM) medium (Gibco, United States) supplemented with 10% fetal bovine serum (FBS) (Gibco, United States), and 1% penicillin–streptomycin (100 μg/mL penicillin and 100 μg/mL streptomycin, ThermoFisher). All cells were cultured at 37 °C with 5% CO_2_ in a humidified incubator.

### Antibodies and reagents

The following antibodies were used in western blot: TRIM22 (Proteintech, 13,744-1-AP, 1:1000), p21 (Proteintech, 10,355-1-AP, 1:1000), p53 (Cell Signaling Technology, 9282 T, 1:1000), CDK4 (Proteintech, 11,026-1-AP, 1:1000), CDK6 (Proteintech, 14,052-1-AP, 1:1000), cyclin B1 (Proteintech, 55,004-1-AP, 1:1000), GAPDH (Proteintech, 60,004-1-Ig, 1:3000), Lamin B (Proteintech, 12,987-1-AP, 1:5000), Tubulin (Proteintech, 11,224-1-AP, 1:3000), Vinculin (Proteintech, 66,305-1-Ig, 1:5000), Flag-tag (Sigma-Aldrich, F2555, 1:1000), Myc-tag (Proteintech, 16,286-1-AP, 1:3000), HA-tag (Proteintech, 660,006-2-Ig, 1:2000), Ubiquitin (Proteintech, 10,201-2-AP, 1:2000). The compounds and their sources are as follows: DMSO (Meilun, PWL064), Chloroquine (Selleck, S6999), MG132 (Selleck, S2619), Cycloheximide (Selleck, S7418), Bafilomycin A1 (Selleck, S141310), E-Click EdU Cell Proliferation Imaging Assay Kit (Elabscience, E-CK-A376), Cell Cycle Assay Kit (Elabscience, E-CK-A351), Nuclear and Cytoplasmic Protein Extraction Kit (Beyotime, P0027).

### Cell counting kit-8 (CCK-8) assay

Cell proliferation was estimated by CCK-8 assay, as previously described^44^. Briefly, cells were seeded into 96-well plates at an initial density of 1 × 10^3^ cells/well. Then, CCK-8 reagent (10 μL/well, MA0218, Meilun) was added to each well at the time points of 24, 48, 72, 96, and 120 h post-seeding. After 2 h incubation at 37 °C, we used a microplate reader (Biotek, United States) to obtain the absorbance at 450 nm.

### Colony formation and cell proliferation assay

Cell viability was measured by colony formation and cell proliferation assay^45^. For colony formation assay, cells were seeded into 6-well plates (5 × 10^2^ cells/well) and grown in 10% FBS-supplemented medium for 8–10 days with 3-day intervals of medium renewal. Cells were stained with 0.1% crystal violet (in 10% methanol). Stained colonies were washed 3 times at 10-min intervals, followed by image recording with Tanon 1600 automatic digital image analysis system (Tanontech), and clone numbers analysis with ImageJ software (version 1.54k; https://imagej.net/ij/).

For proliferation assay, cells were seeded into 12-well plates (1 × 10^4^ cells/well) and stained with 0.1% crystal violet at 24-h intervals up to 5 days. After that, we dissolved crystal violet in each well with 10% acetic acid, and the absorbance at 590 nm was detected by the microplate reader (Biotek, United States).

### Edu assay

A 5-ethynyl-2’-deoxyuridine (EdU) assay kit (Elabscience, E-CK-A376) was adopted to assess the cell proliferation ability as described by Fan et al.^46^. According to the manufacturer’s instructions, cells were seeded into confocal plates with a density of 1 × 10^5^ cells/well. On the next day, cells were incubated with 10 μM EdU buffer at 37 °C for 2 h, fixed with 4% formaldehyde for 15 min and permeabilized with 0.1% Triton X-100 for 20 min. EdU solution was added to the culture medium, followed by the staining of nuclei with DAPI (4',6-diamidino-2-phenylindole). Then the cells were examined and counted under a Fluorescence microscope.

### Cell Cycle assay

The cell cycle was assessed using standard protocols^47^. We used a cell cycle assay kit (Elabscience, E-CK-A351) following the manufacturer’s instructions. In brief, 5 × 10^5^ cells were washed with PBS and then fixed in 1.5 mL of pre-cooled 80% ethanol for 1 h at −20 °C. After centrifugation at 300 g for 5 min, the cells were resuspended in 1 mL PBS and then incubated at 37 °C for 15 min. Then, the cells were centrifuged at 300 g for 5 min and resuspended in 100 μL RNase A Reagent. After incubation at 37 °C for 30 min, the cells were resuspended in 400 μL propidium iodide solution (50 μg/mL). Subsequently, the cells were incubated in the dark environment at 2–8 °C for 30 min and then analyzed by flow cytometry.

### Subcellular fractionation

Extraction and isolation of nuclear and cytoplasmic proteins from A375 cells were performed by the Nuclear and Cytoplasmic Protein Extraction Kit (Beyotime, P0027). The protocols for protein extraction from the cytoplasm and nucleus were in accordance with the Kit instructions.

### Western blot

Cells were harvested with the cell scraper and cellular protein was extracted using RIPA lysis buffer [50 mM Tris–Cl (pH7.4), 150 mM NaCl, 1 mM EDTA (pH8.0), 0.25% DOC (deoxycholic acid), 10% glycerol, 1% Nonidet P-40 and 1% Triton-X100] with 1% protease inhibitor cocktail (Selleck, B14001) and 1% phosphatase inhibitor cocktail (Selleck, B15001). The lysates were centrifuged at 12,000 g for 15 min at 4 °C and the supernatant was collected. The total protein concentration was quantified using BCA protein assay kit (Thermo Fisher Scientific A65453). Protein expression was determined using western blotting. Briefly, after SDS-PAGE (Sodium Dodecyl Sulfate–Polyacrylamide Gel Electrophoresis), the proteins were transferred to polyvinylidene difluoride (PVDF) membranes (Millipore). The membranes were blocked with 5% skim milk for 60 min at room temperature, and then incubated overnight with specific primary antibodies at 4 °C. After recycling the primary antibodies on the next day, the membranes were washed with TBST Buffer (150 mM NaCl, 20 mM Tris–Cl, 1% TWEEN-20, pH7.4) and subsequently incubated with secondary antibodies (1:1000, Beyotime, Nanjing, China) for 2 h at room temperature. The membranes were then developed using SuperSignal™ West Pico PLUS ECL Substrate (Thermo Fisher Scientific 34,580).

### RNA extraction, reverse-transcription and quantitative real-time PCR (qRT-PCR)

Total RNA was extracted from cells with RNAiso Plus (Takara 9109) reagent and reverse-transcribed to cDNA with ALL-in-One First-Strand Synthesis MasterMix (with dsDNase). qRT-PCR was performed using SYBR GREEN (Yeasen 11201ES08) according to the manufacturer’s protocol with CFX-96 instrument (Bio-Rad). Sequences of primer pairs were as follows:

- TRIM22
- F 5’- GAGGTCAAGATGAGCCCACAG -3’
- R 5’- GCTTTTCCTGACATTCCTTGACC -3’
- P21
- F 5’- AGGCACTCAGAGGAGGTGAG -3’
- R 5’- CCGCAGAAACACCTGTGAA -3’
- GAPDH
- F 5’- GGACCTGACCTG CCGTCTAG -3’
- R 5’- GTAGCCCAGGATGCCCTTGA -3’

### Plasmid construction

Full-length and truncated TRIM22 coding sequences were amplified by PCR and inserted into the pCDNA3.1 or pBOBI vector with HA, Flag, or Myc tag at appropriate restriction endonuclease sites. To establish stable TRIM22 or p21 gene-silencing cell lines, a lentiviral short hairpin RNA (shRNA) technique was used. Specific shRNA sequences were cloned into the pLKO.1 vector at the AgeI/EcoRI sites at the 3’ end of the human U6 promoter. The sequences used were shown as follows:

- shTRIM22#1
- F 5’- CCGGTCGACCTGCTTATCCGTATTTCTCGAGAAATACGGATAAGCAGGTCGATTTTTG -3’
- R 5’- AATTCAAAAATCGACCTGCTTATCCGTATTTCTCGAGAAATACGGATAAGCAGGTCGA -3’
- shTRIM22#3
- F-5’- CCGGGTCACCAAACATTCCGCATAACTCGAGTTATGCGGAATGTTTGGTGACTTTTTG -3’
- R 5’- AATTCAAAAAGTCACCAAACATTCCGCATAACTCGAGTTATGCGGAATGTTTGGTGAC -3’
- shp21#1
- F 5’- CCGGCGCTCTACATCTTCTGCCTTACTCGAGTAAGGCAGAAGATGTAGAGCGTTTTTG -3’
- R 5’- AATTCAAAAACGCTCTACATCTTCTGCCTTACTCGAGTAAGGCAGAAGATGTAGAGCG -3’

### Transfection and lentivirus infection

HEK293T cells were used for transfection and lentivirus packaging. Plasmids and PEI (Polyethylenimine, Polysciences 24,765) were mixed at 1:4 weight-to-weight (w/w) ratio in Opti-MEM medium (Gibco), and then added to 293 T cell medium. Protein expression was tested about 48 h after transfection.

For lentivirus packaging, the plasmids (control and shRNA-expressing lentivirus vectors) were transfected into 293 T cells together with packaging vectors psPAX2 and pVSVG using PEI. Virus-containing medium was harvested 48 h post-transfection, filtered with 0.45 μm membrane to remove cell debris, and then used to culture target cells for 8–24 h. Positive cells were selected using puromycin or blasticidin, depending on the lentivirus vector resistance gene.

### Clinical specimens

Twelve malignant melanoma samples were obtained from The First Affiliated Hospital of Dalian Medical University. The eligible samples were pathologically diagnosed as malignant melanoma between 2019 and 2022. Tumor staging was defined according to the 8th Edition of the American Joint Committee on Cancer (AJCC8) Staging Manual. All tissues were formalin-fixed paraffin-embedded tissues. No patient received preoperative treatment, and all patients or their proxies provided written or verbal informed consent to participate in this study. The methods of this study were reported in accordance with the Declaration of Helsinki. The protocol used in this study was approved by the Institutional Ethics Committee, IRB No. PJ-KS-KY-2024-129.

### Immunohistochemistry (IHC)

IHC was performed to detect the expression of TRIM22 in paraffin sections of melanoma tissues. The tissues were sectioned into 4 μm slides for IHC. The slides were deparaffinized, hydrated and incubated with rabbit polyclonal anti-TRIM22 (Proteintech, 1:600) antibody at 4 °C overnight, followed by incubation for 1 h with goat anti-rabbit IgG-HRP (IgG-Horseradish Peroxidase) and subsequent visualization. DAB (3,3'-Diaminobenzidine, Proteintech, China) was used as chromogen. IHC staining was evaluated using ImageJ. We ensured consistency by maintaining the same setting for all the analyzed fields. Integrated optical density (IOD) was used to assess the staining intensity.

### S-protein pull-down assay

Cells expressing SF-tagged protein were lysed with NETN buffer [20 mM Tris–Cl (pH7.4), 100 mM NaCl, 1 mM EDTA (pH8.0), 0.5% Nonidet P-40] followed by centrifugation to obtain the supernatant. The cell lysates were then incubated with S-protein agarose (Millipore) at 4 °C for 4 h. The agarose was washed 4 times with NETN buffer and 1 × SDS-PAGE loading buffer was added to the agarose. The proteins bound to the agarose were released by thermal denaturation and then subjected to Western blot.

### Public database analysis

The expression of TRIM22 at mRNA level in both Cancer Genome Atlas (TCGA; https://portal.gdc.cancer.gov/) and Genotype-Tissue Expression (GTEx; https://gtexportal.org/home/) samples were analyzed using the Gene Expression Profiling Interactive Analysis 2 (GEPIA2; http://gepia2.cancer-pku.cn/), Tumor IMmune Estimation Resource 2.0 (TIMER2.0; http://timer.cistrome.org/) and the TNMplot databases (http://www.tnmplot.com/). To validate the findings based on TCGA, we further utilized datasets GSE98394 from the Gene Expression Omnibus (GEO; https://www.ncbi.nlm.nih.gov/gds/), providing an independent validation set for our results. Using the Human Protein Atlas (HPA; https://www.proteinatlas.org/) database, we further assessed the local expression pattern of TRIM22 in tissue samples. The correlation between TRIM22 expression and TRIM22 target genes was evaluated using the GeneMANIA online software (version 3.6.0; http://genemania.org/).

### Statistical analysis

Similar results were obtained in at least three independent experiments. Data were expressed as mean $\pm$ Standard deviation (SD). $\chi$^2^-test was employed to compare qualitative variables. Analysis of quantitative variables was performed using the *T* test or one-way analysis of variance (ANOVA). A *p* value < 0.05 was considered statistically significant. All statistical analyzes were performed using the GraphPad Prism 9.0. *p < 0.05, **p < 0.01, ***p < 0.001.

## Results

### TRIM22 is upregulated in melanoma and associated with clinical features

**Fig. 1:**
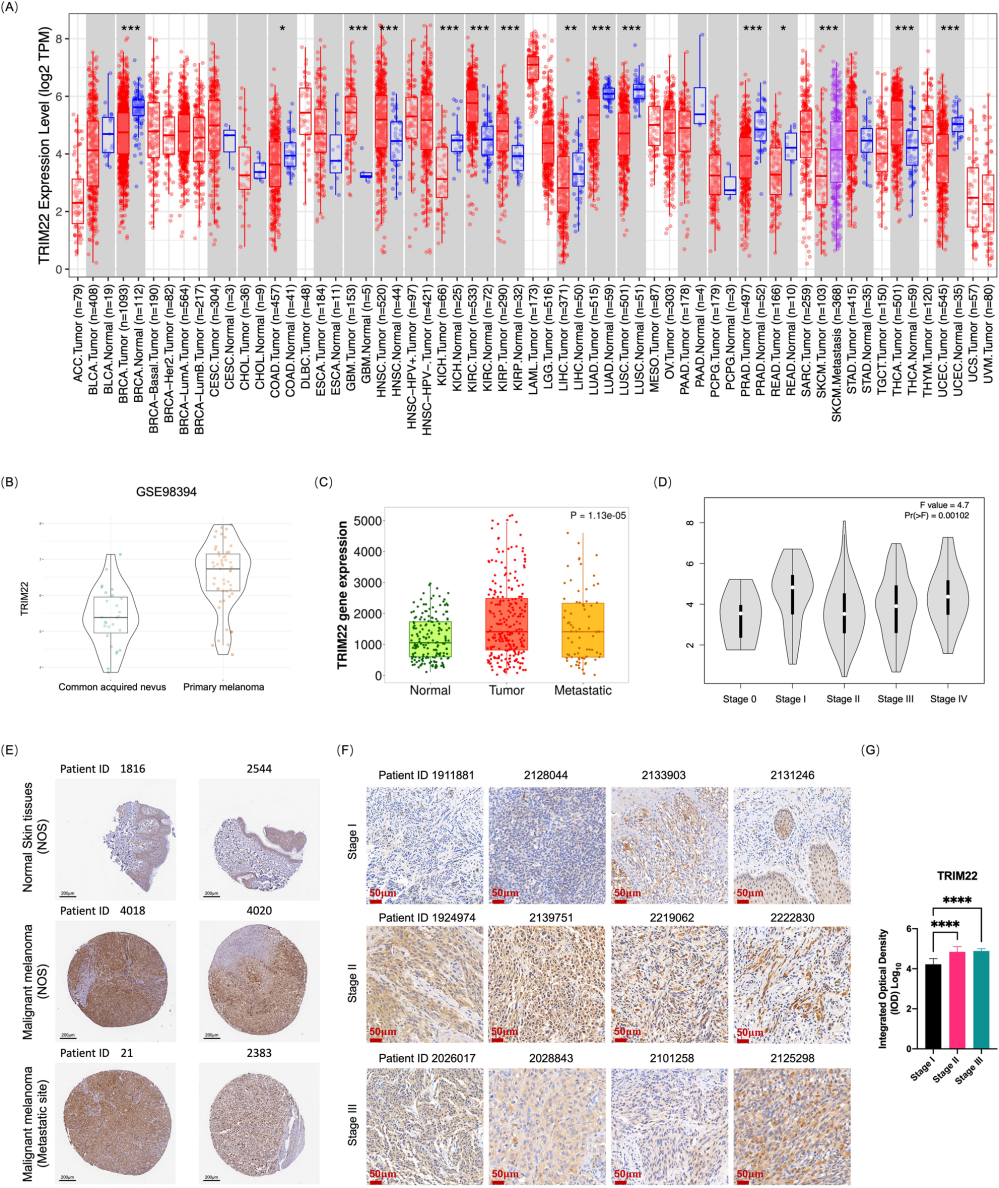
The TRIM22 expression in human SKCM tissues and its correlation with clinical characteristics. (**A**) Analysis of TRIM22 gene expression at the RNA level across various cancer types, providing insights into the transcriptomic landscape (TIMER2.0). ***p < 0.0001. (**B**) TRIM22 mRNA expressions in GSE98394 dataset of the GEO database. Mann–Whitney U test: *p* = 2.29 × 10^−6^. (**C**) TNMplot data for the expression of TRIM22 gene in skin tumors (*n* = 253), normal (*n* = 174), and metastatic tissue (*n* = 76). Kruskal–Wallis test: *p* = 1.13 × 10^−5^. (**D**) Correlation between the expression of TRIM22 and tumor stage in SKCM patients (GEPIA2). (**E**) Representative IHC micrographs of TRIM22 in skin samples and melanoma from the HPA database. Not Otherwise Specified (NOS). Scale bar, 200 μm. (**F**) Representative IHC micrographs of TRIM22 in collected clinical samples of melanoma samples. Quantification of the integrated optical density (IOD) is presented in the corresponding graph. *N* = 4 in each group. Scale bar, 50 μm. Data were shown as Mean ± SD. The standard deviations of independent experiments were represented by error bars. One-way ANOVA. ****p < 0.0001.

To determine the expression of TRIM22 in melanoma and its clinical implications, we first analyzed TRIM22 pan-cancer mRNA expression levels using the TCGA database and the TIMER2.0 tool^48^. We found that TRIM22 show varied expression across different cancer types, with significantly higher expression in GBM, head and neck squamous cell carcinoma (HNSC), kidney renal clear cell carcinoma (KIRC), kidney renal papillary cell carcinoma (KIRP), metastatic skin cutaneous melanoma (SKCM), and thyroid carcinoma (THCA) (Fig. 1A). To compare melanoma expression with common acquired nervus we used datasets from the GEO database (GSE98394) and observed that the expression of TRIM22 was significantly higher in primary melanoma than in common acquired nevus (*p* < 0.0001) (Fig. 1B). We further compared normal, tumor and metastatic tissues with TNMplot^49^ and as shown in Fig. 1C, TRIM22 show remarkably higher expression in skin tumor tissues and metastatic skin tumor tissues compared to normal skin tissues (Fig. 1C). More so, TRIM22 expression was significantly associated with the tumor stage in melanoma samples (Fig. 1D)^50^. To confirm the upregulated expression of TRIM22 in melanoma at protein level, we utilized IHC data from the HPA database. The results also showed that the expression of TRIM22 in melanoma tissue was significantly higher compared to normal skin tissues (Fig. 1E). Further investigation of IHC from clinical melanoma patients revealed significantly elevated expression of TRIM22 in stage II and stage III tumors (Fig. 1F, G), corroborating our initial finding of a direct association of TRIM22 with tumor stage. In general, these results verified the overexpression of TRIM22 in melanoma and its possible implication as a potential biomarker in melanoma progression.

### TRIM22 promotes melanoma cell proliferation in vitro

**Fig. 2:**
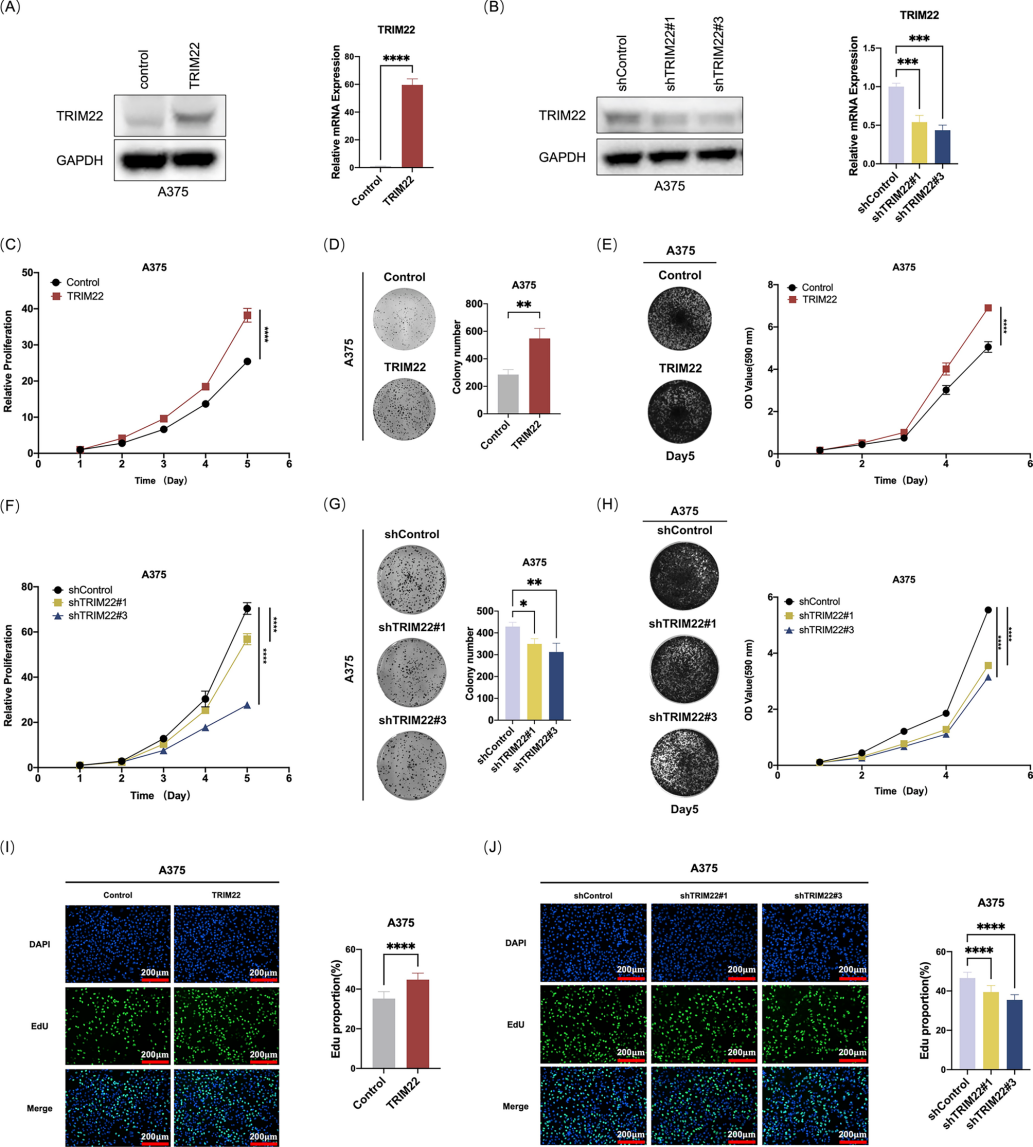
TRIM22 promotes melanoma cell proliferation in vitro. (**A**) Western blots and qRT-PCR were performed to characterize the expression of TRIM22 in the control and TRIM22-overexpression A375 cells. (**B**) Western blots and qRT-PCR were performed to characterize the expression of TRIM22 in the control and TRIM22-knockdown A375 cells. (**C**–**E**) CCK-8 (**C**), colony formation (**D**) and colony growth proliferation (**E**) assays were performed to evaluate the growth ability of A375 cells with or without TRIM22 overexpression. (**F**–**H**) CCK-8 (**C**), colony formation (**D**) and colony growth proliferation (**E**) assays were performed to evaluate the growth ability of A375 cells with or without TRIM22 konckdown. (**I**–**J**) EdU assays were performed to evaluate the growth ability of the control, TRIM22-overexpression, and TRIM22-knockdown A375 cells. Scale bar, 200 μm. Quantitative analysis was performed using ImageJ software. All data were expressed as the mean ± SD. The standard deviations of independent experiments were represented by error bars. The data were analyzed by two-tailed unpaired t-test or One-way ANOVA. *p < 0.05, **p < 0.01, ***p < 0.001, ****p < 0.0001.

Next, we explore the impact of TRIM22 on the viability of melanoma cells. We begin with the construction of stably TRIM22 overexpressed and knocked down A375 cell lines, which were validated by western blotting and qRT-PCR (Fig. 2A, B). Subsequently, we investigated the effect of TRIM22 on melanoma cell proliferation via different methods, including the CCK-8 assay, crystal violet staining, colonogenic assay and EdU assay. With classical CCK-8 and crystal violet staining, we observed a direct correlation between TRIM22 expression and A375 cell proliferation. As shown in Fig. 2, overexpression of TRIM22 significantly increased the proliferation of A375 cells (Fig. 2C and E), while knockdown of TRIM22 decreased the cell proliferation (Fig. 2F and H). Similarly, our colony formation assay, confirmed that the proliferation ability of A375 cells, indicated by the number of clones was elevated by TRIM22 overexpression (Fig. 2D) and was substantially decreased with TRIM22 knockdown (Fig. 2G). Finally, we employed EdU assays to measure cell viability by DNA quantification, and the results further proved that TRIM22 significantly enhanced the proliferation of A375 cells (Fig. 2I, J). Collectively, these results provide compelling evidence that TRIM22 can promote melanoma proliferation in vitro.

### TRIM22 promotes melanoma cell cycle progression and decreases p21 expression

**Fig. 3:**
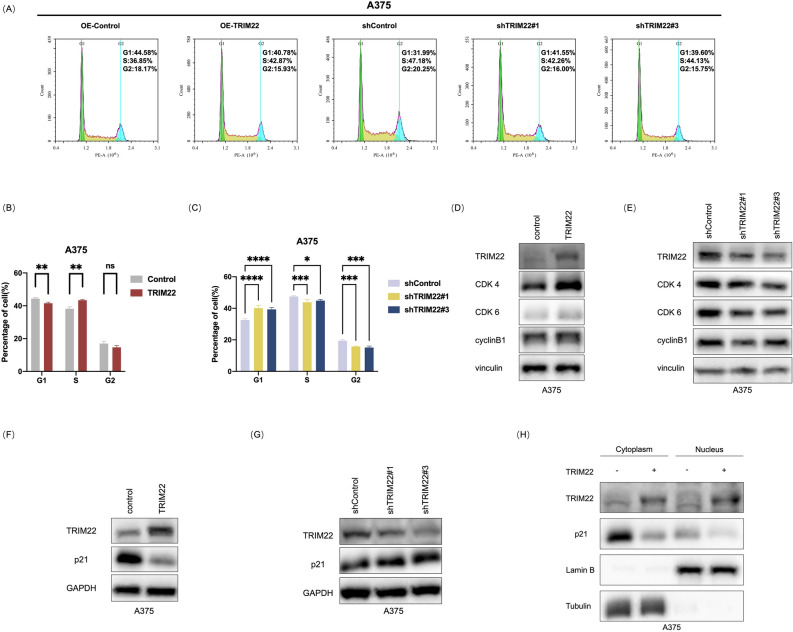
TRIM22 regulates the cell cycle progression of melanoma cells and decreases p21 expression. (**A**) Flow cytometry assays were performed to evaluate the cell cycle distributions in TRIM22-overexpression and TRIM22-knockdown A375 cells. (**B**–**C**) The cell cycle was analyzed in TRIM22 overexpression (**B**) and knockdown (**C**) A375 cells by flow cytometry assay. (**D**–**E**) Western blots were performed to characterize the expression of CDK4, CDK6, and cyclinB1 in TRIM22 overexpression (**D**) and knockdown (**E**) A375 cells. (**F**–**G**) Western blots were performed to characterize the expression of p21 in TRIM22overexpression (**F**) and knockdown (**G**) A375 cells. (**H**) Relative protein levels of p21 in the cytoplasm and nucleus of the control and TRIM22-overexpression A375 cells assayed by nucleocytoplasmic separation. All data were expressed as the mean ± SD. The standard deviations of independent experiments were represented by error bars. The data were analyzed by two-tailed unpaired t-test or One-way ANOVA. *p < 0.05, **p < 0.01, ***p < 0.001, ****p < 0.0001.

To investigate the mechanism by which TRIM22 promotes melanoma proliferation, we used flow cytometry to determine the effect of TRIM22 on the cell cycle progression, as cell cycle is a critical event for proliferation. Interestingly, we found that TRIM22 overexpression resulted in an increase in the ratio of S-phase cells and a decrease in the ratio of G1-phase cells (Fig. 3A and B). Conversely, TRIM22 knockdown causes G1 phase cell arrest and subsequent reduction of the S phase cells (Fig. 3A and C). Given this finding, we proceeded to examine the levels of cell cycle proteins in TRIM22 overexpressed and knockdown cells. Consistently, we found upregulation of cyclin-dependent kinases 4 (CDK4), cyclin-dependent kinases 6 (CDK6), and cyclin B1 in TRIM22 overexpression cells (Fig. 3D), and their downregulation in TRIM22 knockdown cells (Fig. 3E), indicating that TRIM22 could regulate cell cycle proteins. As p21 plays a key role in the cell cycle progression^51^, we speculated that it may be involved in the TRIM22-mediated melanoma proliferation. Therefore, we further examined the expression of p21 protein and found that the p21 protein level was significantly downregulated with TRIM22 overexpression (Fig. 3F) and upregulated with TRIM22 knockdown (Fig. 3G). To further understand the effect of TRIM22 on p21 subcellular distribution, we conducted a nuclear-cytoplasmic separation experiment, and the results illustrated that the reduction of p21 protein was more notable in the cytoplasm of TRIM22 overexpressing cells (Fig. 3H). Taken together, these results demonstrated that TRIM22 could promote melanoma cell proliferation via regulating the cell cycle progression. Moreover, these results also showed a significant negative correlation between the protein levels of TRIM22 and p21 in melanoma cells, indicating a regulatory relationship between these two proteins.

### TRIM22 promotes the degradation of p21 via 26S proteasome in melanoma

**Fig. 4:**
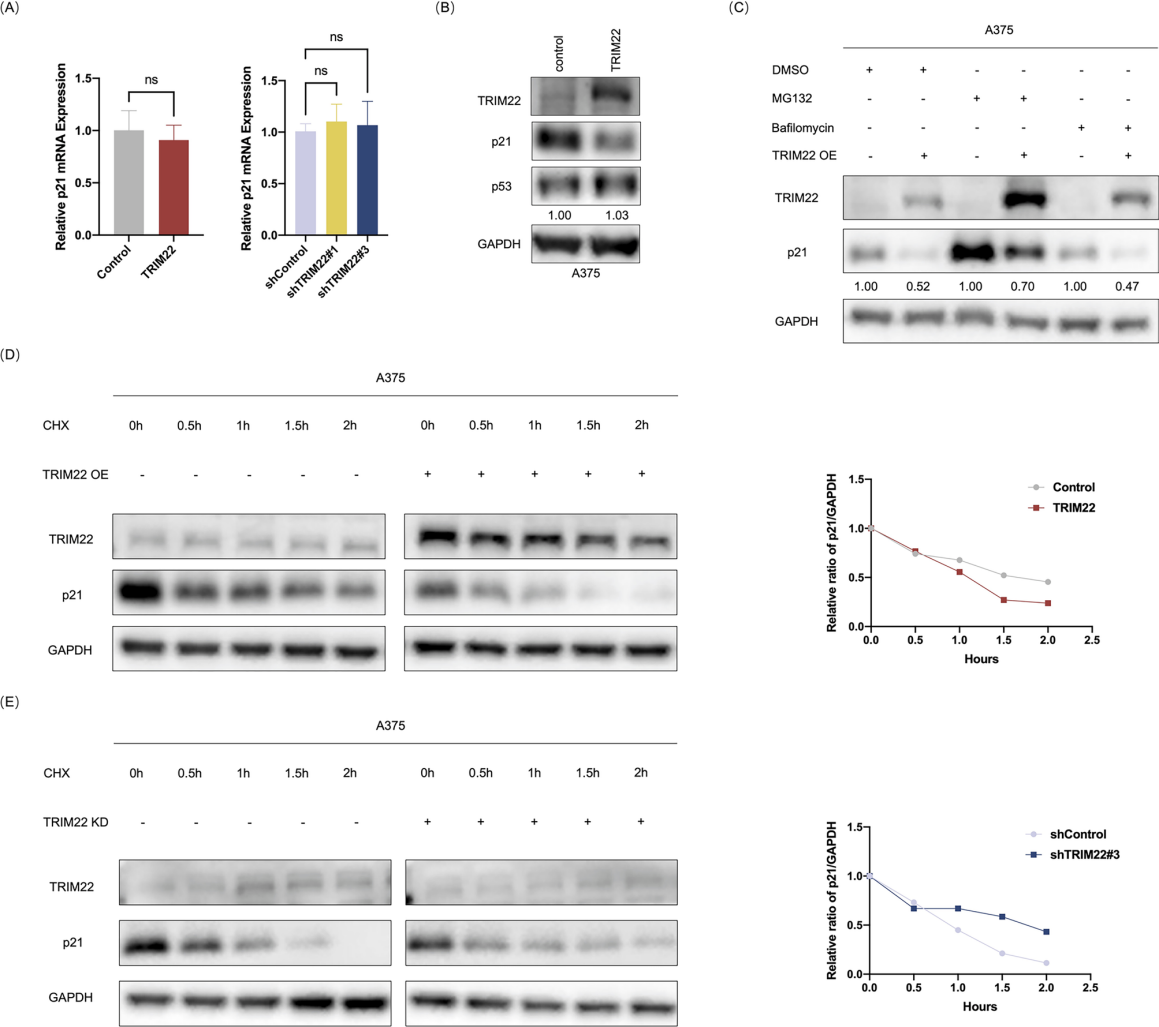
TRIM22 promotes the degradation of p21 protein by ubiquitination in melanoma. (**A**) Relative mRNA levels of p21 in TRIM22-overexpression and TRIM22-knockdown A375 cells assayed by qRT-PCR. All data were expressed as the mean ± SD. The standard deviations of independent experiments were represented by error bars. The data were analyzed by two-tailed unpaired t-test or One-way ANOVA. (**B**) Western blots were performed to characterize the expression of p53 in the control and TRIM22-overexpression A375 cells. (**C**) Western blots were performed to characterize the expression of p21 in TRIM22-overexpression A375 cells treated with DMSO, MG132 and Bafilomycin. (**D**–**E**) Western blots were performed to characterize the expression of p21 in TRIM22overexpression (**D**) and knockdown (**E**) A375 cells treated with CHX for indicated time. Quantification of the p21 levels according to GAPDH were displayed at the right.

The TRIM family is one of the largest classes of RING-finger E3 ligases and participates in a diverse range of cellular signaling transductions and biological processes^29^. With several reports of the implication of TRIM22 in the regulation of protein trafficking, stability, and expression^39,41,52^, it is assumed that TRIM22 could regulate the stability and expression of p21. First, we sought to test the possibility that TRIM22 modulates p21 expression at transcriptional or post-transcriptional level. The first evidence obtained by qRT-PCR analyses surprisingly revealed that neither overexpression nor knockdown of TRIM22 had an impact on the p21 mRNA level (Fig. 4A). Furthermore, we assessed the expression of p53, a well-known transcriptional regulator of p21 in the cell cycle^51,53^. We tested the protein level of p53 in TRIM22 overexpression cells and found that it did not also correlate with TRIM22 level (Fig. 4B). These findings exclude the possibility of TRIM22 regulation of p21 at the transcription or post-transcriptional level, implying that TRIM22 could regulate p21 at the protein level and possibly via post translational modification.

Given that TRIM22 is an E3 ligase associated with inducing proteasome degradation of its target proteins, we next investigated whether TRIM22 promoted p21 degradation through the canonical ubiquitin-26S proteasome pathway. As shown in Fig. 4C, TRIM22 overexpression downregulated p21 and this downregulation was dramatically reversed by MG132 (carbobenzoxyl-L-leucyl-L-leucyl-L-leucine), a proteasome inhibitor, despite high accumulation of TRIM22. On the other hand, the autophagy inhibitor Bafilomycin exhibited no obvious effect on p21 levels (Fig. 4C). Based on the above findings, we further sought to investigate the effect of TRIM22 on the protein stability of endogenous p21 using the protein synthesis inhibitor, cycloheximide (CHX). The result showed that TRIM22 overexpression markedly promoted p21 degradation (Fig. 4D), whereas the degradation of p21 was inhibited in TRIM22 knockdown cells (Fig. 4E). Together, these results indicate that TRIM22 regulates p21 protein levels by promoting its degradation via 26S proteasome pathway.

### TRIM22 promotes K63-linked ubiquitination of p21

**Fig. 5:**
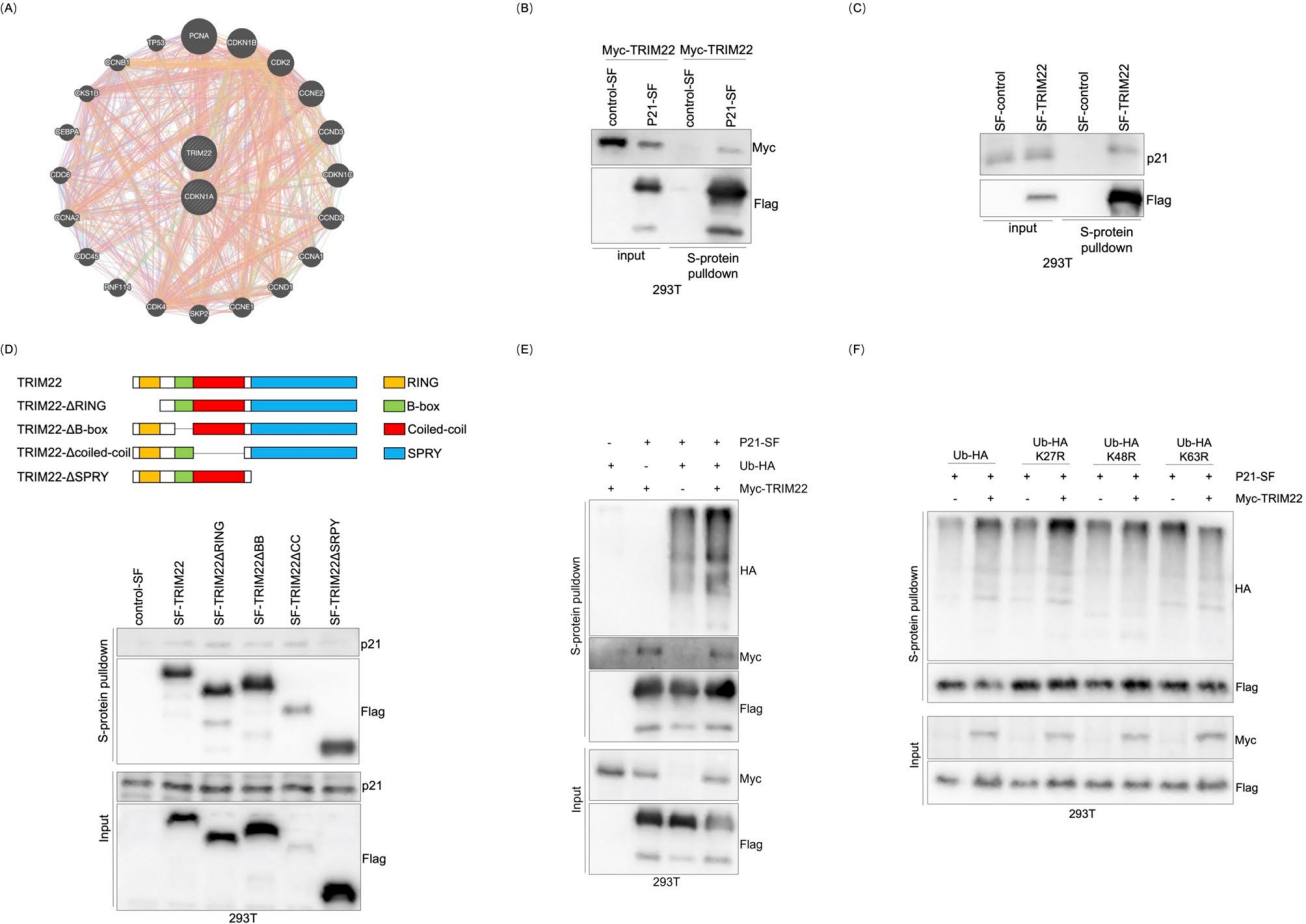
TRIM22 ubiquitinastes p21 and promotes K63-linked ubiquitination of p21. (**A**) GeneMANIA online software was used to analyze the interaction between TRIM22 and p21. (**B**) HEK293T cells were transfected with p21-SF and Myc-TRIM22, the cell lysates were immunoprecipitated with anti-Myc and anti-Flag antibodies, respectively. Then, anti-Myc and anti-Flag antibodies were used to detect the immunoprecipitates, and exogenous interactions of TRIM22 and p21 were tested by immunoblotting. (**C**) HEK293T cells were transfected with SF-TRIM22, the cell lysates were immunoprecipitated with anti-p21 and anti-Flag antibodies, and endogenous interactions of TRIM22 and p21 were tested by immunoblotting. (**D**) SF-tag full-length and truncated TRIM22 constructs were transfected into HEK293T cells, lysates were pulled-down with S-protein agarose to elucidate the interacting domain of TRIM22 to p21. The schematic representation of TRIM22 deletion mutants(up), and the interaction between p21 and TRIM22 truncations (below). (**E**) Ubiquitination assay of p21 in HEK293T cells co-transfected with p21-SF, MYC-TRIM22, and UB-HA, and the transfected 293T cells were treated with MG132 before proteins were harvested. Then the cells were lysed and pulled-down with S-protein agarose, and the indicated protein levels were tested by immunoblotting. (**F**) Ubiquitination assay of p21 in HEK293T cells co-transfected with Myc-TRIM22, p21-SF, UbHA, or K27R-HA, K48R-HA, and K63R-HA with the treatment of MG132. Then the cells were lysed and pulled-down with S-protein agarose, and the indicated protein levels were tested by immunoblotting.

To explore the potential mechanism by which TRIM22 regulates p21 degradation in melanoma, we established a comprehensive gene interaction network involving TRIM22 and p21, leveraging the GeneMANIA database, and detected a predicted interaction between TRIM22 and p21 (Fig. 5A)^54^. To verify this interaction, we performed an S-protein pull-down assay in 293 T cells. The results indicated that Myc-tagged TRIM22 interacted with SF-tagged (S-protein tag and Flag tag) p21 (Fig. 5B), suggesting a physical association between the two proteins. Then, we performed a pull-down assay in 293 T cells transiently expressing SF-tagged TRIM22, and the results confirmed that endogenous p21 interacted with TRIM22 (Fig. 5C). To investigate the functional domains of TRIM22 responsible for the interaction with p21, we expressed a series of domain truncated mutants of TRIM22 in 293 T cells and performed an S-protein pull-down assay. The result showed that the SPRY domain of TRIM22 was responsible for its interaction with p21 (Fig. 5D). Given that most of the TRIM family members function as E3 ligases^29^, we hypothesized that TRIM22-induced p21 degradation might be link to its E3 ligase-mediated protein ubiquitination. Thus, we co-expressed Myc-TRIM22, p21-SF, Ub-HA and control vectors in 293T cells, followed by the S-protein pull-down assay to identify whether TRIM22 could attach the polyubiquitin chain to p21. Remarkably, TRIM22 led to an increase in the polyubiquitination of p21 (Fig. 5E). To further determine the type of p21 ubiquitination mediated by TRIM22, we used mutant ubiquitin in which lysine (K) at positions 27, 48, or 63 was replaced by arginine (R), respectively. The result identified that TRIM22 promoted K63-linked ubiquitination of p21 (Fig. 5F). Together, these data suggest that TRIM22 regulates p21 stability by acting as an E3 ligase and inducing p21 K63-linked ubiquitination.

### TRIM22 regulates melanoma cell proliferation through p21

**Fig. 6:**
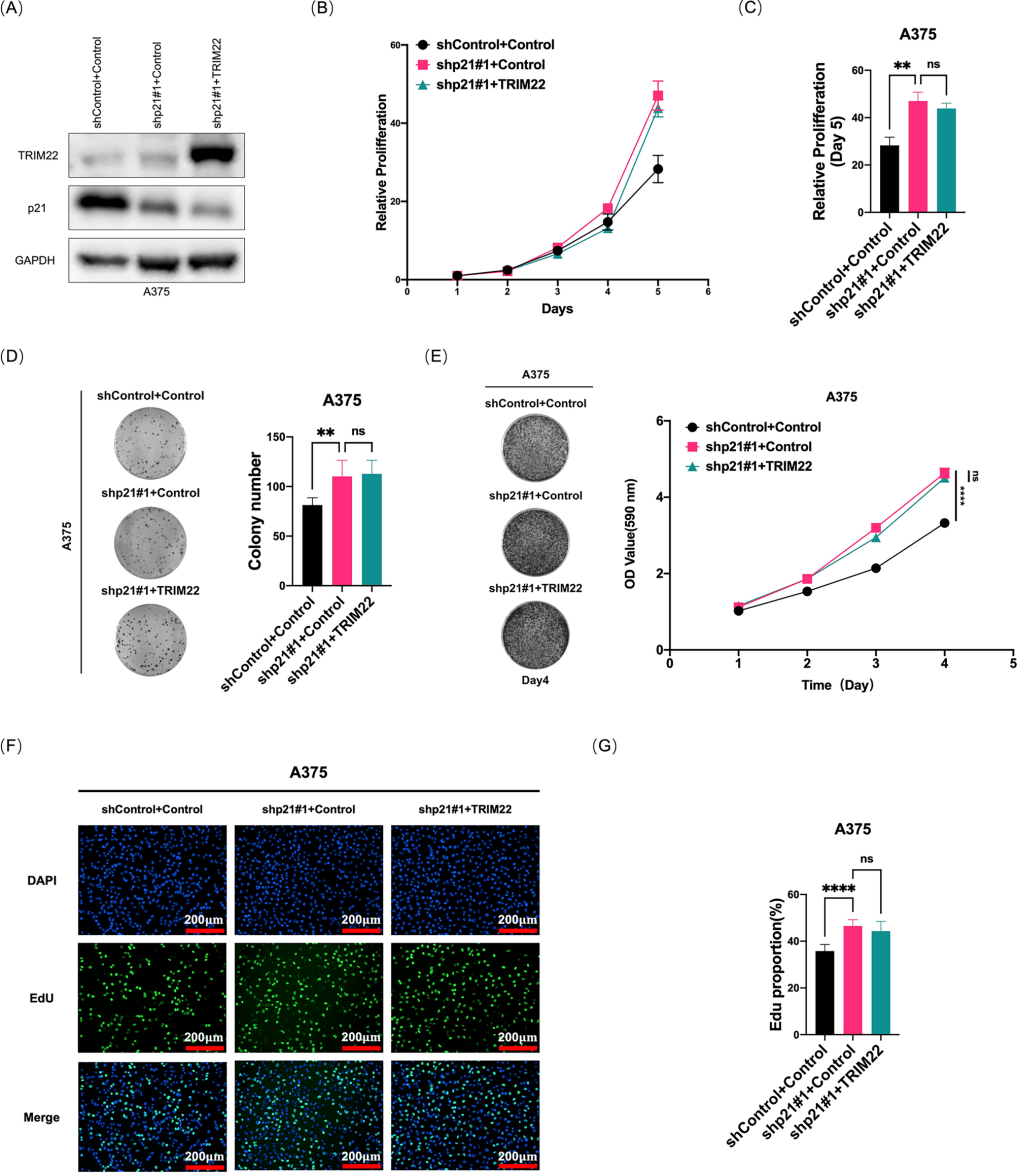
p21 mediates the melanoma proliferation effects of TRIM22. (**A**) Western blots were performed to characterize the expression of TRIM22 and p21 in the shControl, shp21, and shp21 + TRIM22 A375 cells. (**B**–**C**) CCK-8 assays were performed to evaluate the growth ability of shControl, shp21, shp21 + TRIM22 A375 cells. (**D**–**E**) Colony formation and colony growth proliferation assays were performed to evaluate the growth ability of shControl, shp21, shp21 + TRIM22 A375 cells. (**F**–**G**) EdU assays were performed to evaluate the growth ability of the control, shControl, shp21, shp21 + TRIM22 A375 cells. Scale bar, 200 μm. Quantitative analysis was performed using ImageJ software. All data were expressed as the mean ± SD. The standard deviations of independent experiments were represented by error bars. The data were analyzed by two-tailed unpaired t-test or One-way ANOVA. *p < 0.05, **p < 0.01, ***p < 0.001, ****p < 0.0001.

Our data have demonstrated that TRIM22 directly interacts with p21 for ubiquitination and regulates the stability of p21. To further determine whether TRIM22 exerts the role of promoting melanoma cell proliferation through p21, we overexpressed TRIM22 in p21-knockdown A375 cells (Fig. 6A) and investigated the impact on the proliferation of melanoma cells. We used CCK-8 assays (Fig. 6B, C), colony formation assays (Fig. 6D), crystal violet staining (Fig. 6E), and EdU assays (Fig. 6F, G) to evaluate the cell proliferation as was done previously. As shown in Fig. 6B–G, knockdown of p21 resulted in accelerated cell proliferation, while overexpressing TRIM22 in the p21 knockdown cells could not induce any further elevation of the cell proliferation ability, as it had achieved in wild-type A375 cells (Fig. 2C, D, E and I). The reason could be that, as TRIM22 promoted cell proliferation at least partially through decreasing p21 level, the overexpression of TRIM22 in p21 knockdown cells could not cause any further p21 reduction (Fig. 6A), hence no further cell proliferation boost (Fig. 6B–G). Taken together, these results clarified that TRIM22 regulates the proliferation of melanoma cells at least partially through targeting its substrate p21 for degradation to promote cell cycle progression.

## Discussion

Ubiquitination, an important type of PTM, plays a crucial role in regulating protein functions. It is intricately associated with the development and progression of diseases, particularly cancer^55–57^. Several studies have identified the critical roles of ubiquitination in the development, progression, and therapeutic resistance of melanoma^19,58^. TRIM22, a member of the TRIM family, acts as an E3 ligase in the initiation and progression of various malignant tumors. However, the role of TRIM22 in melanoma remains unclear; hence, we aim to investigate the expression and functional relevance of TRIM22 in melanoma.

**Fig. 7:**
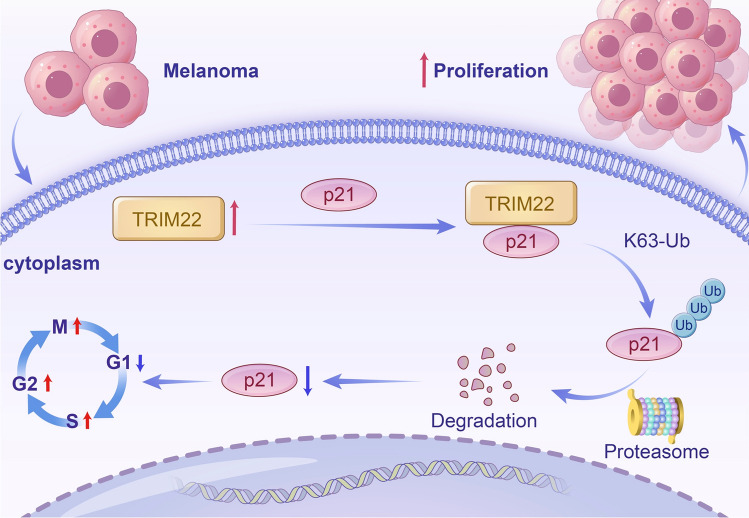
A model for TRIM22 ubiquitinates p21 and thus promotes melanoma cell proliferation.

Beginning with the analysis of public databases and clinical samples, we found that TRIM22 was highly upregulated in melanoma and its expression level was correlated with the clinical stage (Fig. 1A–G). Such significantly high expression suggests a functional role in tumor progression; thus, we performed a viability assay to assess to role of TRIM22 in melanoma cell proliferation. Our result revealed that TRIM22 overexpression promoted the proliferation of melanoma cells while the knockdown inhibited melanoma cell proliferation (Fig. 2A–J), implying that the high expression of TRIM22 observed in public and clinical data studies could be linked to melanoma progression. Mechanistically, overexpression of TRIM22 could promote the proliferation of melanoma cells by accelerating cell cycle progression through degrading p21 via the 26S proteasome system (Fig. 7). Moreover, biochemical assay demonstrated that TRIM22 interacted with p21 and promoted its K63-linked ubiquitination. These findings highlight the important role of TRIM22 in melanoma progression.

Melanoma, as one of the most heterogeneous cancers, displays a high level of biological complexity during disease progression^59–61^, warranting critical investigation of protein regulations that influence its malignant features. Although TRIM family members exhibit diverse functions in different types of human cancers, their roles in melanoma have received little attention. TRIM14 has been shown to promote melanoma malignancy via the PTEN/PI3K/AKT and STAT3 pathways^62^. TRIM15 facilitates ERK1/2 activation by K63-linked polyubiquitination in melanoma^63^. More so, high expression of TRIM27 is significantly associated with shorter disease-free survival and overall survival, positioning it as a novel biomarker for melanoma^64^. For TRIM22, studies show that it plays a contrasting role in different cancers. For instance, in GBM, TRIM22 plays an important role in promoting GBM proliferation and chemoresistance to temozolomide^38,39,65^, while in cancers like hepatocellular carcinoma (HCC), gastric cancer (GC), and BC^42,66,67^, TRIM22 functions as a tumor suppressor. Given these varying functions of TRIM22 in different cancers and limited investigation in melanoma, there is a need for a clear understanding of the role of TRIM22 in melanoma and the underlying regulatory mechanism. Accordingly, our findings provide new insight into the function and involvement of TRIM22 in melanoma, which would contribute to the overall understanding of TRIM family function in tumors.

The RING domain constitutes the catalytic center of TRIMs, and it exerts its role by mediating protein–protein interactions. Structure–function evaluations demonstrated that most TRIM family members contain a RING domain and function as an E3 ligase^31^. In non-small cell lung cancer, TRIM72 interacts with Ras GTPase-activating protein SH3 domain-binding protein 2 (G3BP2) through the RING domain^68^. TRIM22 contains an N-terminal RING-finger domain, a B-box domain, a coiled-coil domain, a cos-box domain, and a C-terminal SPRY domain (Fig. 5D)^29,69^. Several studies have reported that TRIM22 mediates ubiquitination of target proteins through its RING domain^39,70^. However, not all TRIM proteins function as an E3 ligase through the RING domain. For instance, the RING domain of TRIM56 can directly interact with cellular inhibitor of apoptosis protein-1 (cIAP1), causing its deubiquitylation in GBM^71^. It has been reported that the B-box domain of TRIM45 is important for the interaction with receptor for activated C kinase 1 (RACK1) in cancer cells^72^. TRIM29, lacking the RING domain, acts as an E3 ligase responsible for mediating ubiquitination of insulin-like growth factor-2 mRNA-binding protein 2 (IGF2BP1) through the C-terminal domain^73^. Meanwhile, TRIM65 has been shown to bind neurofibromatosis type 2 (NF2) and mediate the ubiquitylation of NF2 at the K44 residue by its SPRY domain^74^. The SPRY domain of TRIM25 is the key structural domain governing the K63-linked ubiquitination of non-POU domain-containing octamer-binding protein (NONO) in GBM^75^. In pancreatic cancer cells, the RING domain and SPRY domain of TRIM15 are both required for the ubiquitination and degradation of Apolipoprotein A1 (APOA1)^76^. Our study demonstrated that TRIM22 interacted with p21 through its SPRY domain (Fig. 5D) and catalyzed the K63-linked polyubiquitination of p21 (Fig. 5F), making it interesting to elucidate the precise mechanism and implication of this modification in further study. Consistent with previous reports, these results further consolidated the function of TRIM22 as an E3 ligase in tumor progression, including melanoma. Additionally, it is suggested that members of the TRIM family may interact with target proteins through different domains and exert functions other than an E3 ligase. Hence, further extensive research is needed to investigate the functions of the TRIM family in various types of cancer, to identify other TRIM22 substrates and to dissect how these interactions affect the physiological function of the target protein.

p21 belongs to the Cip and Kip family of cyclin-dependent kinase (CDK) inhibitors. The *CDKN1A* gene, which encodes the p21 protein, was first discovered as a transcriptional target of p53^77,78^. Although now multiple studies have reported that p21 can be stimulated by many other pathways independent of p53^79^. Recent works suggest that ubiquitination is a critical PTM in p21 regulation and subsequent tumor progression. For instance, F-box only protein 22 (FBXO22) targets p21 for ubiquitin-mediated degradation via the E3 ligase complex in the progression of HCC^80^. Additionally, Ariadne homolog 2 (ARIH2) functions as an E3 ligase of p21 and promotes the proliferation of GC by inducing p21 ubiquitination^81^. Similarly, TRIM52 mediates HCC cell proliferation, invasion, and migration by inhibiting p21 and Mg^2+^/Mn^2+^-dependent 1A (PPM1A) expression^82^. Herein, we discovered that TRIM22 promoted the proliferation of melanoma cells (Fig. 2C–J) which might be linked to its regulation of cell cycle progression (Fig. 3A–C). We examined the expression of cell cycle proteins and found that TRIM22 regulated p21 via K63-linked ubiquitination (Fig. 5B–F). Given that the regulation of p21 through K63-linked ubiquitination is a characteristic function of E3 ligases, these results suggest that TRIM22 is an E3 ligase of p21 and promotes the proliferation of melanoma cells through the ubiquitination of p21. In this paper, we indicated that the SPRY domain is indispensable in TRIM22-mediated ubiquitination (Fig. 5D). The key site of TRIM22 binding with p21 will be further investigated in our subsequent experiments.

Moreover, the diversity of ubiquitin modifications and ubiquitin binding proteins is the foundation of selectivity of the ubiquitin system and allows the transmission of defined signals in a precise spatial and temporal manner^83^. Proteins that are polyubiquitylated through K48 links between the ubiquitin molecules are recognized and degraded by the 26S proteasome^84^, while K63-linked polyubiquitin conjugates are involved in non-proteasomal pathways, including intracellular signaling, DNA repair, and the endosomal-lysosomal system^18,85^. The function of the TRIM family and the fate of the target proteins are influenced by many factors. It has been reported that TRIM31 induces the K63-linked ubiquitination of p53 via its RING domain, and suppresses the murine double minute 2 (MDM2)-mediated K48-linked ubiquitination of p53 through inhibiting the interaction of MDM2 and p53, leading to p53 stabilization and activation in BC^86^. TRIM21 stimulates K63-linked ubiquitination and subcellular translocation of active $\beta$-catenin from the cytoplasm to the nucleus, and inhibits the activity of $\beta$-catenin by inducing K48-linked ubiquitination of transcriptional intermediary factor 1 $\gamma$ (TIF1$\gamma$)^87^. Therefore, the degradation of p21 by TRIM22-induced K63-linked ubiquitination might require the involvement of additional factors and demands more in-depth and comprehensive research. At the same time, other functions and associated mechanisms of TRIM22 in melanoma warrant further exploration.

Taken together, our results illustrate that TRIM22 functions as a tumor-promoting factor in melanoma and reveal that it promotes cell proliferation by targeting p21 for degradation. We provide a novel mechanism in which TRIM22 regulates p21 by functioning as an E3 ligase and induces K63-linked ubiquitination of p21 to regulate melanoma cell cycle progression. As mentioned above, these results will contribute to the future of this research field in several aspects, including expanding the understanding of TRIM family functions in cancer, bringing new insight for investigating the effect of K63-linked ubiquitination on target proteins, and providing more details about the regulation of p21 as well as cell cycle progression especially in cancer. Based on the above findings, TRIM22 is identified as a tumor-promoting factor in melanoma and is inversely associated with the potential cancer suppressor p21. These facts indicate that targeting TRIM22 may be a promising therapeutic target for the treatment of melanoma patients and the establishment of the TRIM22-p21 axis will become an important foundation in the development of new treatment strategies.

## Supplementary Information

Supplementary Information.

## Data Availability

Data are provided within the manuscript or supplementary information files. The raw data supporting the conclusions of this article will be made available by the authors upon reasonable request.
