# Supplementary figures and images for: E3 ligase TRIM22 promotes melanoma proliferation by regulating cell cycle progression through K63-linked ubiquitination of p21
Source: Sci Rep. 2025 Jul 1;15:22311. doi: 10.1038/s41598-025-06348-4 (PMC12216916; doi:10.1038/s41598-025-06348-4)

Fig.2A

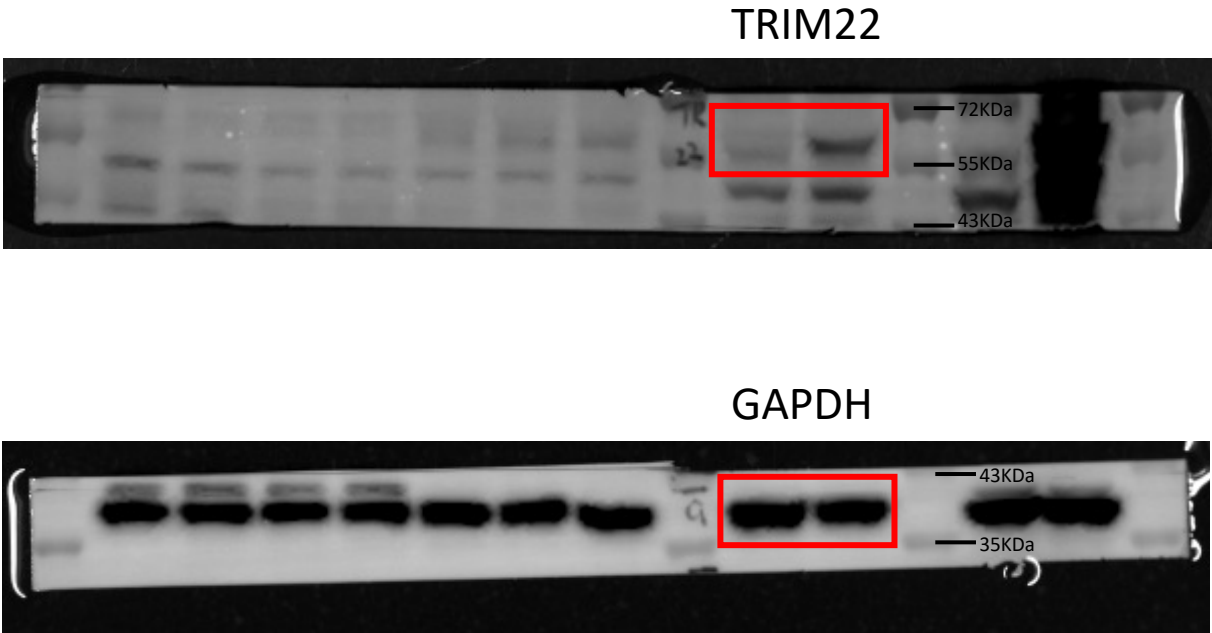

Fig.2B

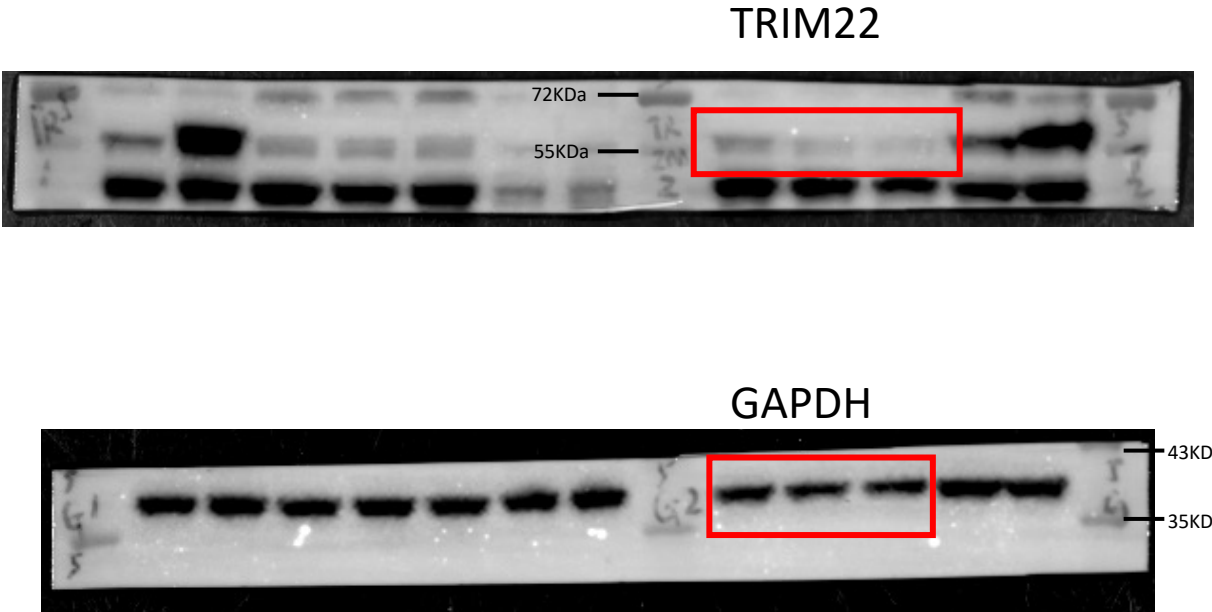

Fig.3D

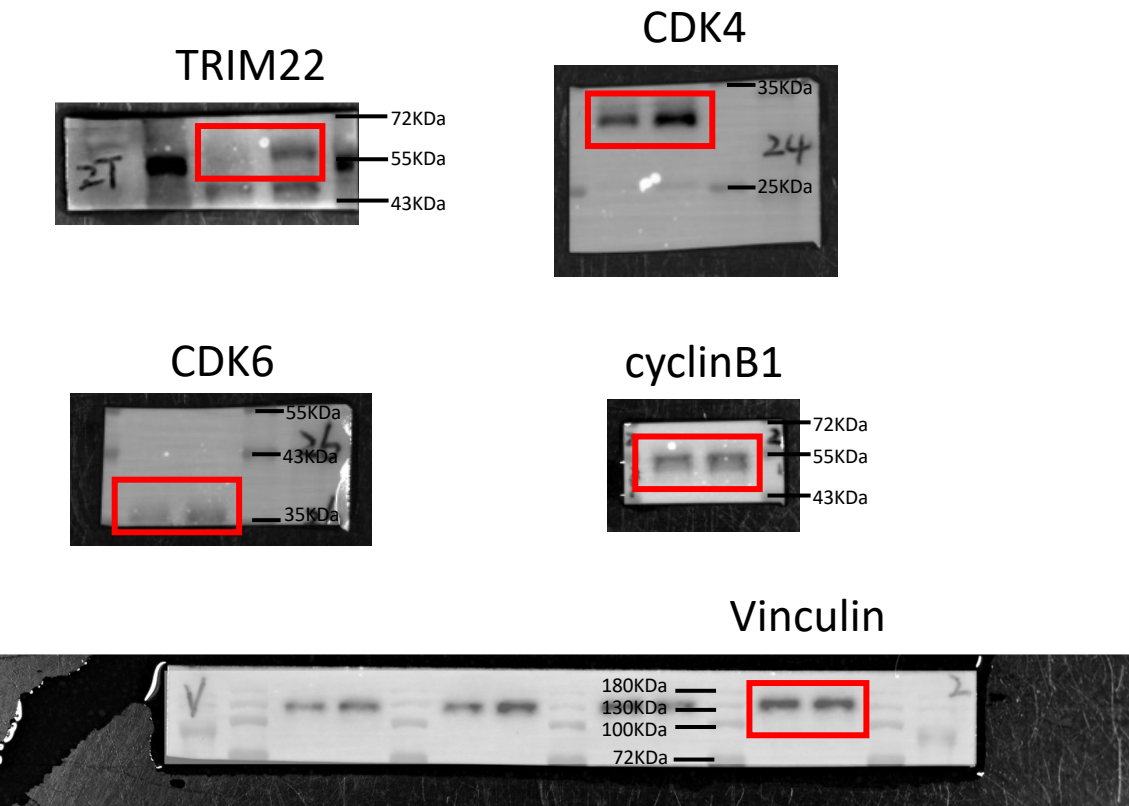

Fig.3E

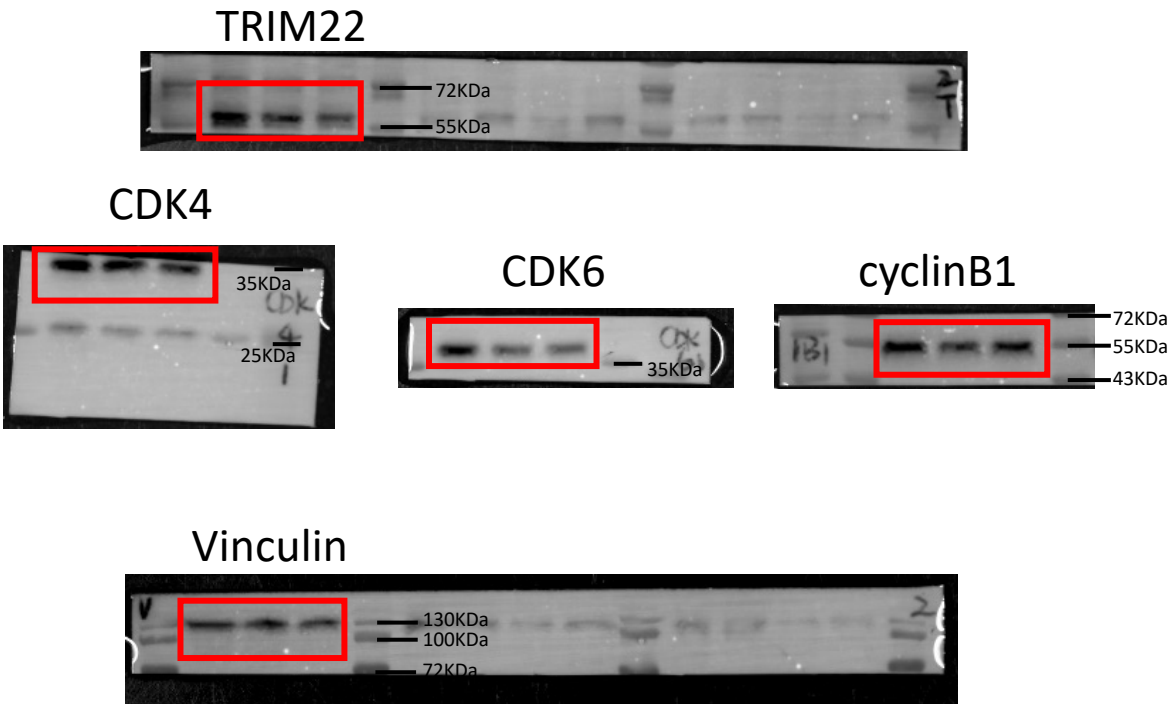

Fig.3F

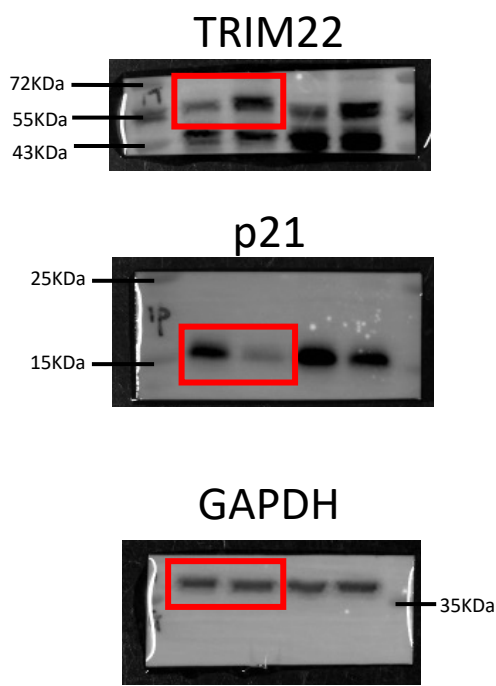

Fig.3G

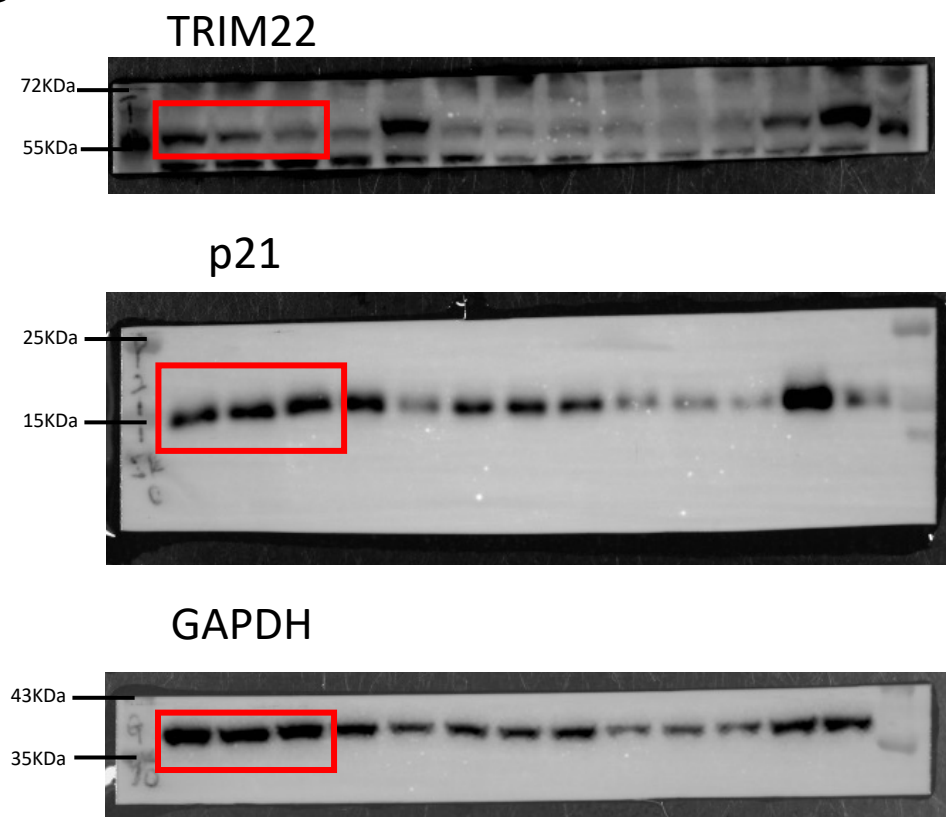

Fig.3H

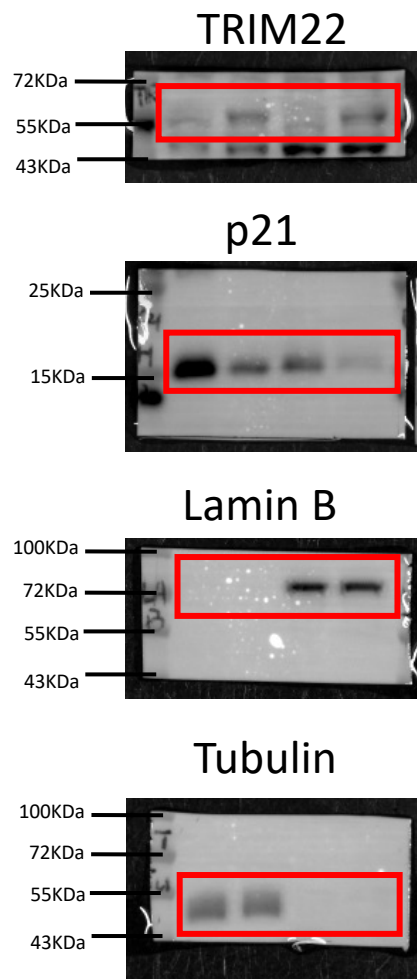

Fig.4B

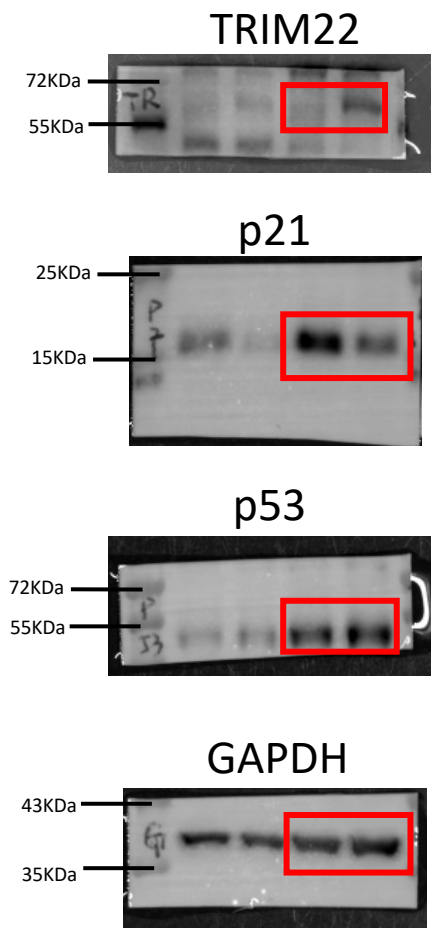

Fig.4C

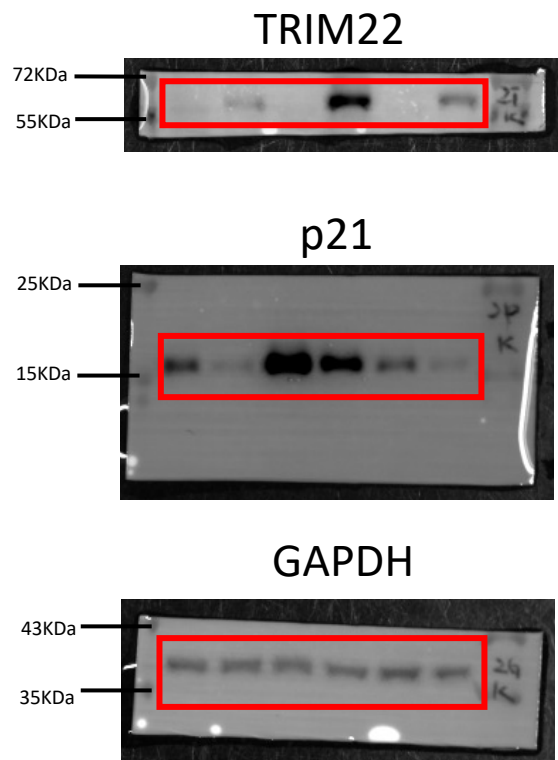

Fig.4D

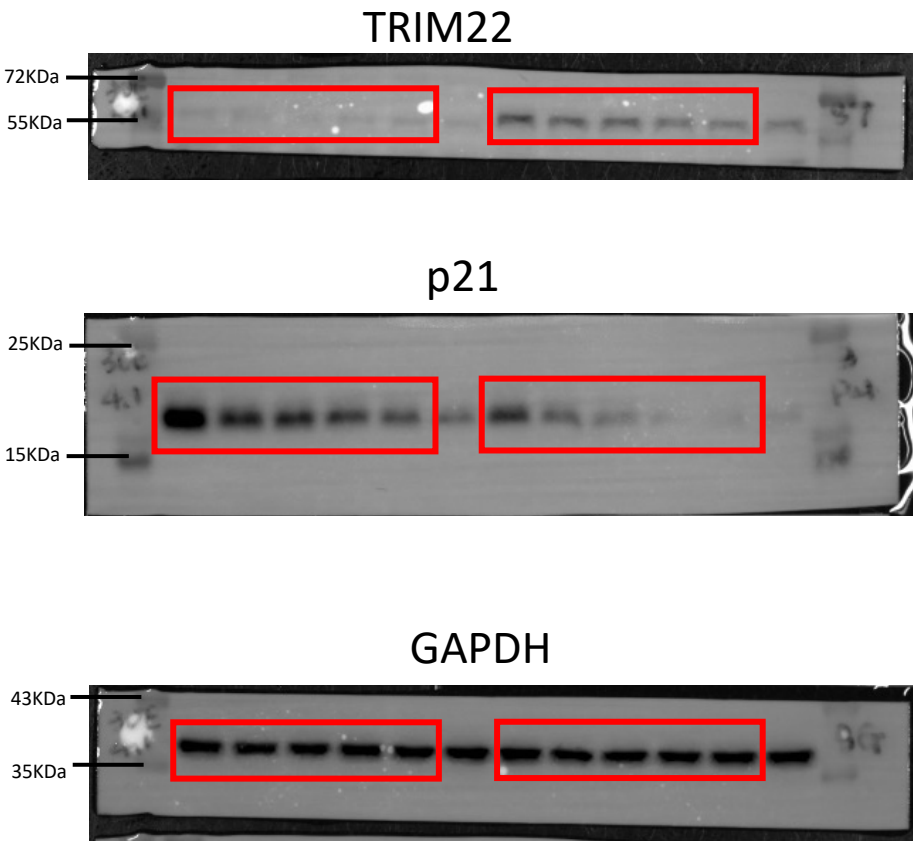

Fig.4E

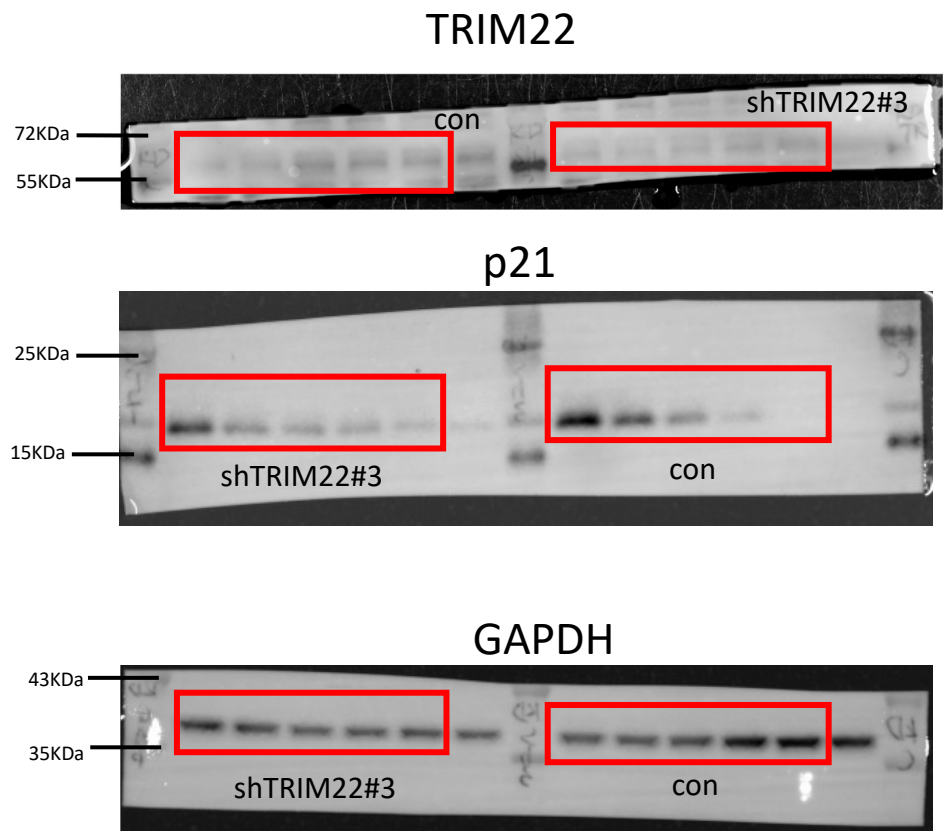

Fig.5B

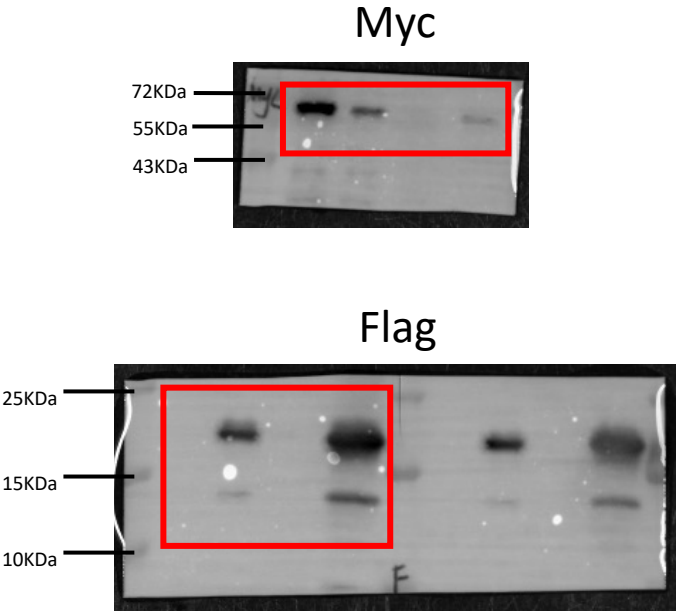

Fig.5C

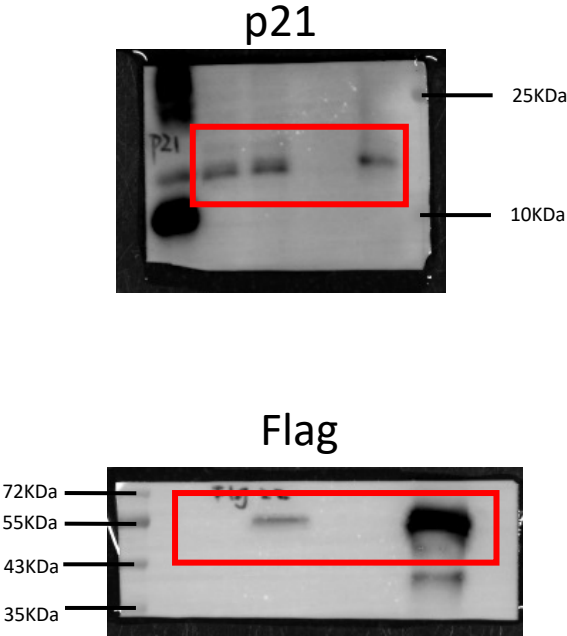

Fig.5D

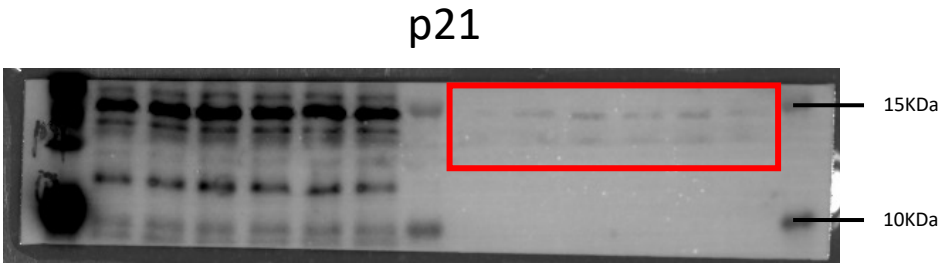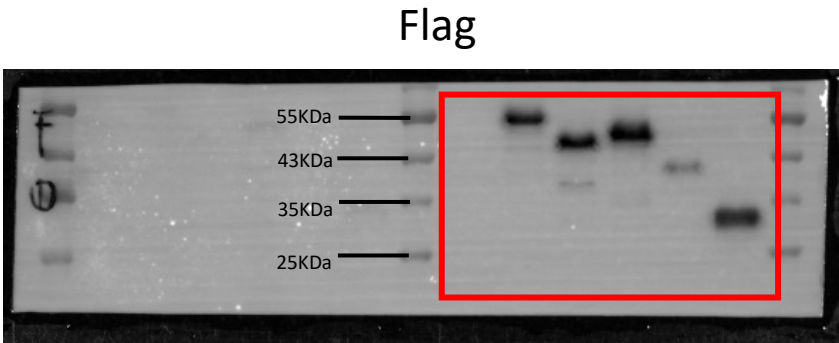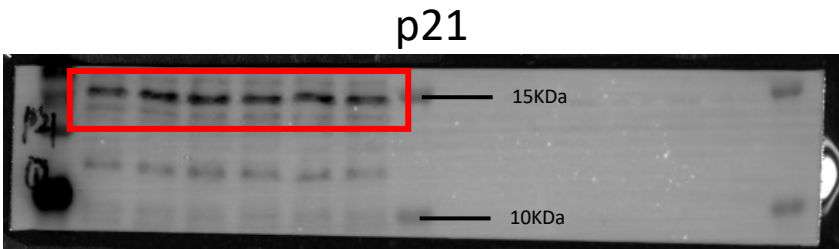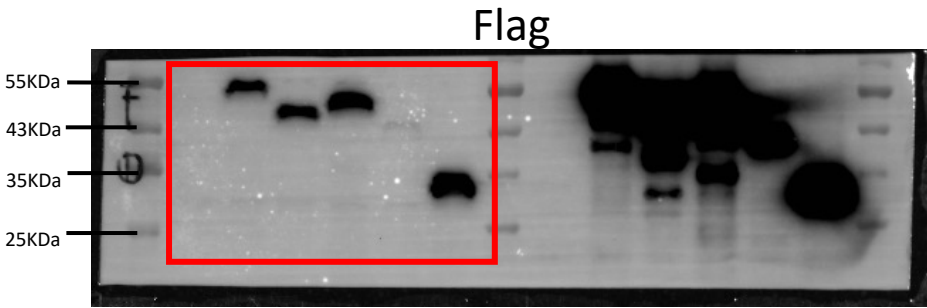

Fig.5E

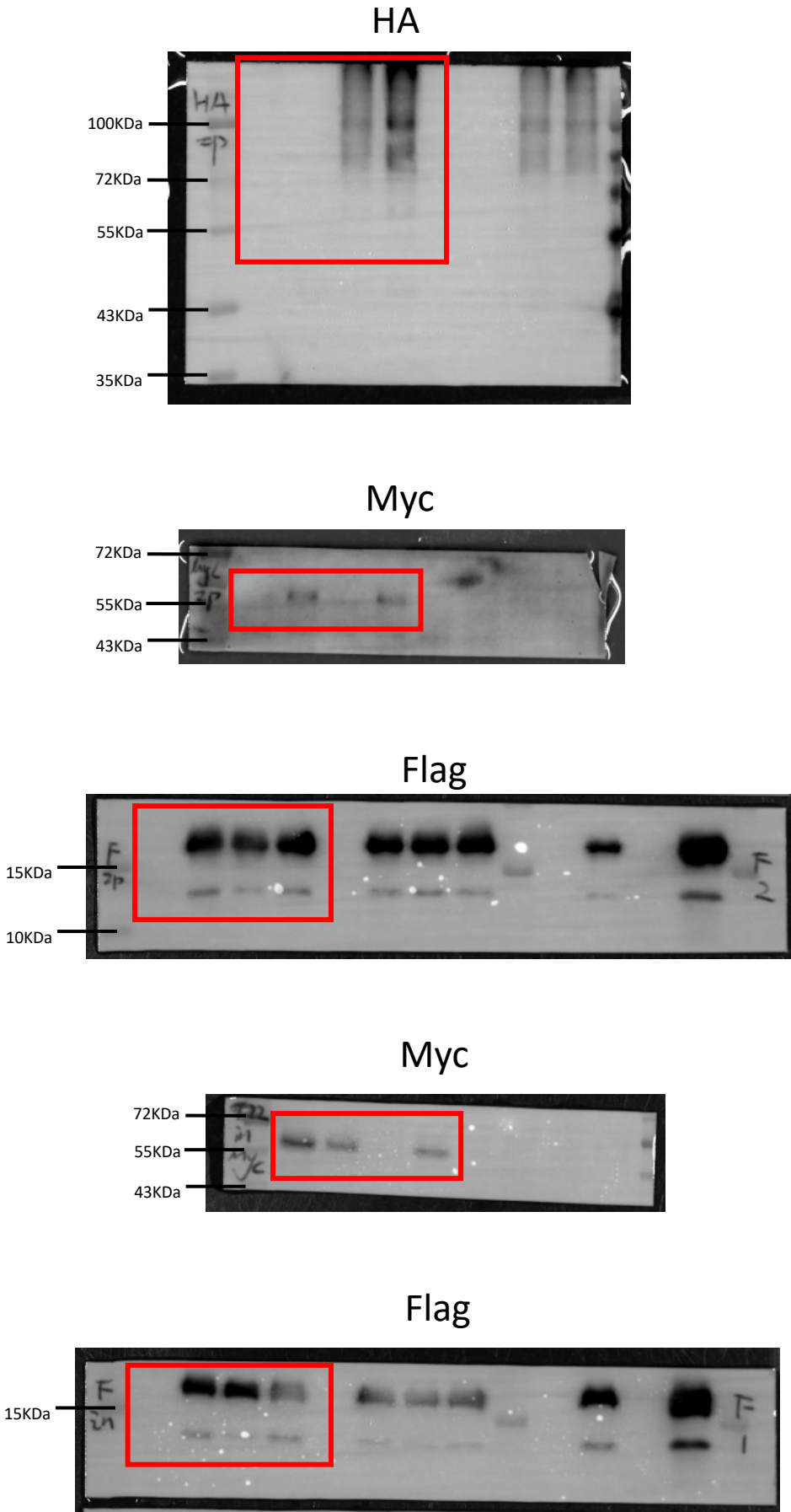

Fig.5F

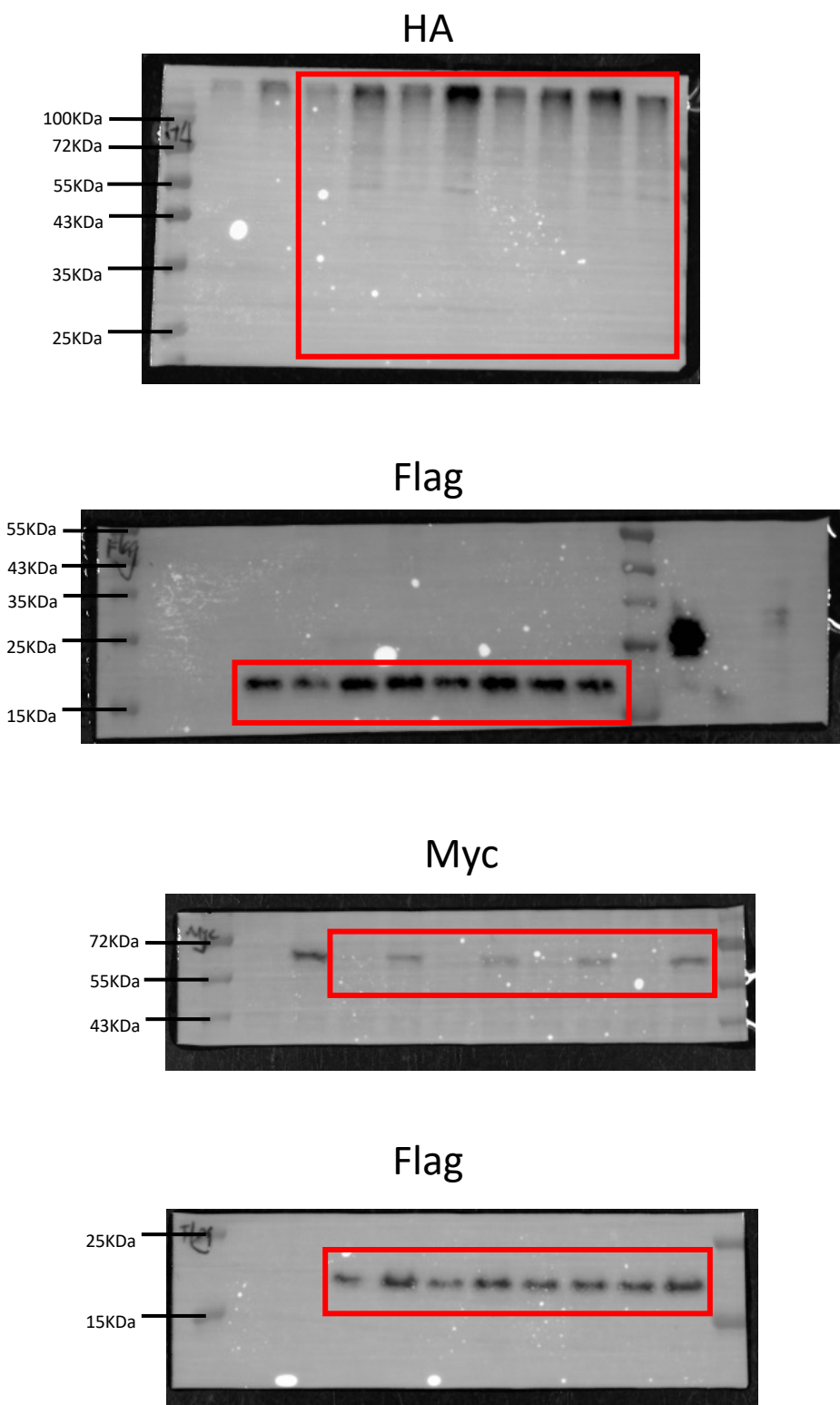

Fig.6A

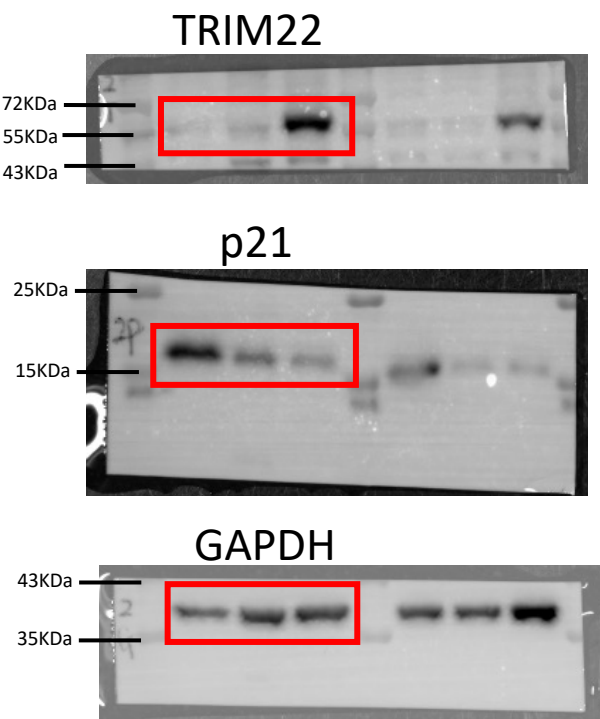

Supplement: Supplementary file 1 — Supplementary Information. [file 41598_2025_6348_MOESM1_ESM.pdf]
